# Induced conformational changes activate the peptidoglycan synthase PBP1B

**DOI:** 10.1111/mmi.14082

**Published:** 2018-10-25

**Authors:** Alexander J. F. Egan, Roberto Maya‐Martinez, Isabel Ayala, Catherine M. Bougault, Manuel Banzhaf, Eefjan Breukink, Waldemar Vollmer, Jean‐Pierre Simorre

**Affiliations:** ^1^ The Centre for Bacterial Cell Biology, Institute for Cell and Molecular Biosciences Newcastle University Richardson Road Newcastle upon Tyne NE2 4AX UK; ^2^ Institut de Biologie Structurale (IBS) Univ. Grenoble Alpes, CNRS, CEA 71 avenue des Martyrs 38000 Grenoble France; ^3^ European Molecular Biology Laboratory Heidelberg, Genome Biology Unit Meyerhofstraße 1 69117 Heidelberg Germany; ^4^ Institute of Microbiology & Infection and School of Biosciences University of Birmingham Edgbaston Birmingham B15 2TT UK; ^5^ Bijvoet Center for Biomolecular Research, Department of Biochemistry of Membranes University of Utrecht Padualaan 8 3584 CH Utrecht The Netherlands

## Abstract

Bacteria surround their cytoplasmic membrane with an essential, stress‐bearing peptidoglycan (PG) layer consisting of glycan chains linked by short peptides into a mesh‐like structure. Growing and dividing cells expand their PG layer using inner‐membrane anchored PG synthases, including Penicillin‐binding proteins (PBPs), which participate in dynamic protein complexes to facilitate cell wall growth. In *Escherichia coli*, and presumably other Gram‐negative bacteria, growth of the mainly single layered PG is regulated by outer membrane‐anchored lipoproteins. The lipoprotein LpoB is required to activate PBP1B, which is a major, bi‐functional PG synthase with glycan chain polymerising (glycosyltransferase) and peptide cross‐linking (transpeptidase) activities. In this work we show how the binding of LpoB to the regulatory UB2H domain of PBP1B activates both activities. Binding induces structural changes in the UB2H domain, which transduce to the two catalytic domains by distinct allosteric pathways. We also show how an additional regulator protein, CpoB, is able to selectively modulate the TPase activation by LpoB without interfering with GTase activation.

## Introduction

The morphology and physical robustness of almost all bacteria are maintained by peptidoglycan (PG), a major component of the cell envelope. In diderm Gram‐negative bacteria such as the model *Escherichia coli* the peptidoglycan forms a mainly single‐layered, mesh‐like sacculus situated within the periplasm (Gan et al., 2008; Vollmer et al., 2008). The PG sacculus encases the cell’s cytoplasmic membrane and is essential for cell survival. Several classes of antibiotics, such as β‐lactams and glycopeptides, exploit this essentiality by targeting the PG synthesis pathways causing cell death (Schneider and Sahl, 2010). The sacculus is itself surrounded by the outer membrane (OM), an asymmetric layer of phospholipids and lipopolysaccharide which provides a crucial permeability barrier for the cell, preventing harmful compounds from gaining access beyond (Nikaido, 2003; Tokuda, 2009; May and Silhavy, 2016). Several abundant proteins anchored in the OM bind to the PG layer, and this tight connection is important for maintaining cell envelope stability (Hantke and Braun, 1973; Godlewska et al., 2009; Egan, 2018). Together the layers of the cell envelope work to protect the cell from both mechanical and chemical insults.

The PG sacculus consists of a repeating disaccharide unit, *N‐*acetylglucosamine (Glc*N*Ac) and *N*‐acetylmuramic acid (Mur*N*Ac), polymerised into glycan chains which are connected to each other by short peptides stemming from Mur*N*Ac (Vollmer et al., 2008). This basic structure is repeated across the entire sacculus creating a mesh made up of a single, continuous macromolecule. In order to grow and divide, bacteria must carefully enlarge this molecule in coordination with the growth of the other key envelope components of the cell (Typas et al., 2012; Egan et al., 2016). Any failure in the synthesis and incorporation of new PG could compromise structural integrity and lead to cell death. As such *E. coli* maintains a repertoire of regulatory elements for the control of its PG synthase enzymes (Typas et al., 2012; Egan et al., 2015; 2016), however, the molecular mechanisms of this regulation are largely unexplored.

Two synthetic activities are required for the insertion of new material into the sacculus during growth and division. Glycosyltransferase (GTase) activity polymerises the precursor lipid II into glycan chains and transpeptidase (TPase) activity links the stem peptides of these chains together and/or to the existing sacculus, thereby incorporating new material allowing for growth (Egan et al., 2016). The major synthases in *E. coli*, responsible for the majority of PG growth, are two class A penicillin‐binding proteins (PBPs), PBP1A and PBP1B. The cell requires at least one of these enzymes for growth (Yousif et al., 1985; Denome et al., 1999). Class A PBPs possess both catalytic activities required for the enlargement of the PG sacculus by virtue of two structurally distinct, but functionally coupled, GTase and TPase domains. Function of the TPase domain requires ongoing activity of the GTase (Born et al., 2006). These enzymes also show minor carboxypeptidase activity through side‐reactions of their TPase function (Egan et al., 2015). *E. coli* also has two monofunctional TPase enzymes, PBP2 and PBP3, which are essential for elongation of the cylindrical rod‐shaped cell during growth, and for cell division, respectively (Bertsche et al., 2006; Banzhaf et al., 2012; Egan et al., 2015). PBP1A interacts with PBP2, working during elongation orchestrated by the cytoplasmic, actin‐like, MreB (Banzhaf et al., 2012). PBP1B interacts with PBP3, working during cell division as part of the divisome complex ultimately controlled by the cytoplasmic tubulin‐homologue FtsZ (Bertsche et al., 2006; Müller et al., 2007; Du and Lutkenhaus, 2017; Leclerq et al., 2017). PBP1A and PBP1B both also interact with OM‐anchored lipoproteins LpoA and LpoB, respectively (Paradis‐Bleau et al., 2010; Typas et al., 2010). In both cases, the interaction of each Lpo with its cognate synthase is absolutely essential for its function in the cell, activating the enzymes' TPase activity (Typas et al., 2010; Lupoli et al., 2014). This revealed a new paradigm in Gram‐negative PG growth regulation, as the synthases are not only regulated from inside the cell by cytoskeletal elements, but also from outside the sacculus, suggesting that the cell adjusts the PG synthesis rate in response to the local pore size in the mesh‐like sacculus (Typas et al., 2010; 2012).

In addition to the two catalytic domains, the PBP1B crystal structure revealed the presence of a small, non‐catalytic domain called UB2H (Sung et al., 2009; King et al., 2016). Analysis of the protein sequence of PBP1A also revealed a similar non‐catalytic domain called ODD (Typas et al., 2010). In fact, numerous class A PBPs across the bacterial kingdom possess such non‐catalytic domains (Typas et al., 2012). It was recently shown that *P. aeruginosa* PBP1B, which has a UB2H domain but no LpoB homologue according to sequence data, has its own unique Lpo protein ‘LpoP’ (Greene et al., 2018). As with the *E. coli* Lpo proteins LpoP is essential for *Pseudomonas* PBP1B function. In order to interact with and regulate PBP1B in *E. coli*, LpoB’s structured domain reaches from its anchor point in the OM by virtue of a 145 Å long disordered sequence to interact directly with UB2H via a relatively large interface (Egan et al., 2014). This interaction stimulated both the GTase and TPase activities of PBP1B. The question nevertheless remained, how does interaction with the non‐catalytic UB2H domain activate both catalytic domains of PBP1B? Interestingly, a genetic screen isolated three PBP1B versions with amino acid substitutions clustering in the inter‐domain region of the synthase, and one in the GTase domain, which partially bypass the need for activation by LpoB (Markovski et al., 2016). This supports a model in which activation of the synthase proceeds via a conformational change initiated by LpoB binding to its UB2H domain (Egan et al., 2014; Markovski et al., 2016). This hypothesis was further supported by the isolation of *P. aeruginosa* PBP1B versions with similar Lpo bypass mutations (Greene et al., 2018). However, the mechanism by which LpoB binding activates both PG synthesis activities simultaneously remained elusive.

An additional layer to the regulation of PBP1B by LpoB was subsequently discovered in *E. coli*. The Tol‐Pal associated protein CpoB interacts with PBP1B and selectively inhibits the LpoB‐mediated activation of TPase, but not GTase, activity in response to Tol‐Pal function in the cell (Gray et al., 2015). Tol‐Pal is implicated in many roles related to OM integrity across several Gram‐negative species, including in OM constriction during cell division (Gerding et al., 2007; Egan, 2018). This selective uncoupling of the two activation effects suggests that CpoB binding blocks only the conformational change affecting the TPase domain and not GTase but this remained to be investigated.

Here, we present the structure of the isolated UB2H domain in solution and confirm the stability of its fold in the absence of the TPase and GTase domains. We show that the binding of LpoB to UB2H induces limited conformational and dynamic changes in the domain, mainly localised at the extremities that are normally linked to the rest of the PBP1B structure both covalently and through hydrogen‐bonding networks. Based on this evidence, we investigated the importance of these hydrogen‐bonding networks which connect UB2H with the TPase and GTase domains, by testing the cellular functionality of PBP1B versions altered in key residues. We then tested the activity of these versions, and their interaction with and activation by LpoB. Our findings show that binding of LpoB to UB2H induces two distinct yet overlapping allosteric activation routes through the domain, impacting the activities of the GTase and TPase domains through localised hydrogen‐bonding reorientation. This reorganisation could structurally explain the activation process of the two catalytic domains and also reveals the importance of structural dynamics in class A PBP activity. Furthermore, we determine the structure of the C‐terminal TetratricoPeptide Repeat (TPR) domain of *E. coli* CpoB, identify the interaction surfaces between this domain and PBP1B, and infer a mechanism by which binding achieves selective inhibition of TPase activation.

## Results

### Binding of LpoB alters the structure of UB2H

**Figure 1:**
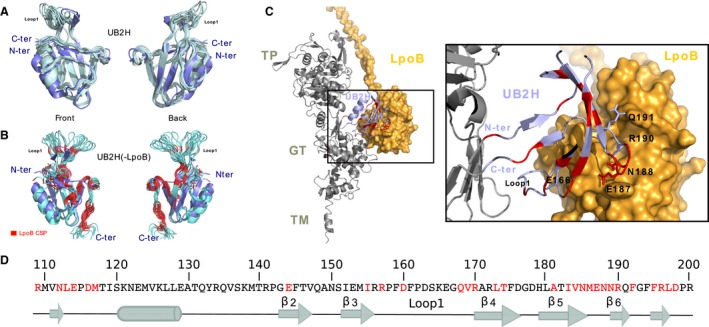
Binding of LpoB induces structural changes in UB2H. Superimposition of the 20 lowest energy structures derived from NMR of A. the isolated UB2H domain (in pale blue, PDB 6FZK) or B. the UB2H domain in complex with LpoB (in blue, PDB 6G5R) and of the X‐ray structure of the UB2H domain in full‐length PBP1B (PDB code 5FGZ, in purple). Residues that showed a significant chemical shift perturbation upon binding of LpoB are shown in red in panel B. C. The same residues are coloured in red on the previously reported model that was obtained from a data‐driven docking of the PBP1B X‐ray structure (PDB 3FWL) and the NMR LpoB structure (PDB 2MII). This model was calculated based on the LpoB CSP induced by UB2H binding and on the identification of few UB2H residues that impacted activation of PBP1B upon *in vivo* mutations (Egan et al., 2014). Labels and sticks for side chains are displayed for the concerned mutated residues. For clarity, the zoom presented in the right panel corresponds to a partial view selected to emphasise the N‐ and C‐terminal extremities of UB2H as well as the loop region 186 to 191 that is important for the interaction with LpoB. The two UB2H extremities (residues 109‐115 and 193‐200) are covalently linked to β‐strands β1 and β7 that form the inter‐domain region of PBP1B. D. Protein sequence of the UB2H domain with residues in red that showed a significant perturbation upon LpoB binding and identification of secondary structure elements in free UB2H. [Colour figure can be viewed at http://www.wileyonlinelibrary.com]

To investigate possible structural changes to the UB2H domain resulting from LpoB binding we expressed and purified the UB2H domain of *E. coli* PBP1B (residues 108 to 200) and determined its structure by NMR spectroscopy. All NMR data recorded pointed towards the presence of a stable fold for the isolated UB2H domain in solution and were used to calculate a high‐resolution structure (see Table S1). Superimposition of the 20 lowest energy structures derived from NMR (PDB 6FZK) with the UB2H domain from the X‐ray crystal structure of full‐length PBP1B (PDB 5FGZ) shows a similar secondary structure organisation with a backbone rmsd of 1.1 Å for residues involved in secondary structures (Fig. 1A). As described for the X‐ray structure (Sung et al., 2009; King et al., 2016), the domain architecture is formed by one α‐helix followed by four long β‐strands (β2 to β5), which are linked by mostly short turns to form an anti‐parallel β‐sheet. Only the turn between stands β3 and β4 forms a larger loop (Loop 1: residues 157 to 168). This loop shows a lower resolution in our NMR structure, which is correlated to an increase in flexibility in solution in this region, as evidenced by ^15^N‐NMR relaxation measurements (R_2_/R_1_ and ^1^H‐^15^N heteronuclear NOE are shown in Fig. S1A). The N‐ and C‐terminal extremities of the UB2H domain interact through two extended anti‐parallel β segments (residues 109‐115 and 193‐200) that are connected to two very short β‐strands, β1 (105‐108) and β7 (202‐204), that form the intermediate region connecting the TP and GT domains in full‐length PBP1B (PDB 5FGZ).

To monitor possible modifications induced by the binding of LpoB, [^13^C,^15^N]‐labelled UB2H was prepared and mixed with an unlabelled LpoB sample (Fig. S1A). The structure of UB2H in complex with LpoB was calculated using the same procedure as used for UB2H alone and deposited in the PDB (PDB 6G5R). Fig. 1B shows the 20 lowest energy structures calculated for UB2H in complex with LpoB. With the exception of the small anti‐parallel β‐sheet formed between the N‐ and C‐terminal extremities (residues 109–115 and 193–200) of UB2H, all of the secondary structures initially present in UB2H alone were conserved in the complex (Fig. S1B) and the set of UB2H structures in the complex with LpoB could be superimposed with a backbone rmsd of 1.2 Å (calculated for residues in secondary structures) to the X‐ray structure of the UB2H domain of PBP1B (PDB 5FGZ).

To determine the relative position of LpoB in complex with UB2H, we searched for intermolecular distances between the two domains in 3D edited‐NOESY and in 2D ^13^C,^15^N‐filtered NOESY. No intermolecular distance was detectable, consistent with the fast dynamics of the complex formation as revealed by chemical shift perturbations measured on LpoB or UB2H (Fig. S1C) and with surface plasmon resonance (SPR) measurements (Egan et al., 2014). To map the interface between both proteins, we measured the NMR chemical shift perturbations (CSP) induced on the amide groups of UB2H (Fig. S1C). Residues that showed CSP larger than two standard deviations on the UB2H domain or that corresponded to completely disappearing resonances are coloured in red in Fig. 1C. The majority of the large CSP observed localise close to the surface of interaction with LpoB in agreement with our previous model (Egan et al., 2014). This initial model was calculated based on NMR data measured exclusively on LpoB and validated with six key UB2H mutations that impacted the LpoB interaction. While the majority of the CSP in UB2H can be explained by a direct proximity with LpoB, other distal changes could result from structural rearrangements upon binding of LpoB. These structural modifications were confirmed by the difference in Chemical Shift Index (CSI) between the free and bound form of UB2H as determined from H_N_, N, C, Cβ and CO resonances (Berjanskii and Wishart, 2005) (Fig. S1B). The destabilisation of the N‐ and C‐terminal β‐sheet induced by LpoB would modify the network of contacts between UB2H and the rest of PBP1B and likely underlies pathways to transfer the activation signal to the catalytic sites.

### Structural changes in UB2H activate PBP1B

**Figure 2:**
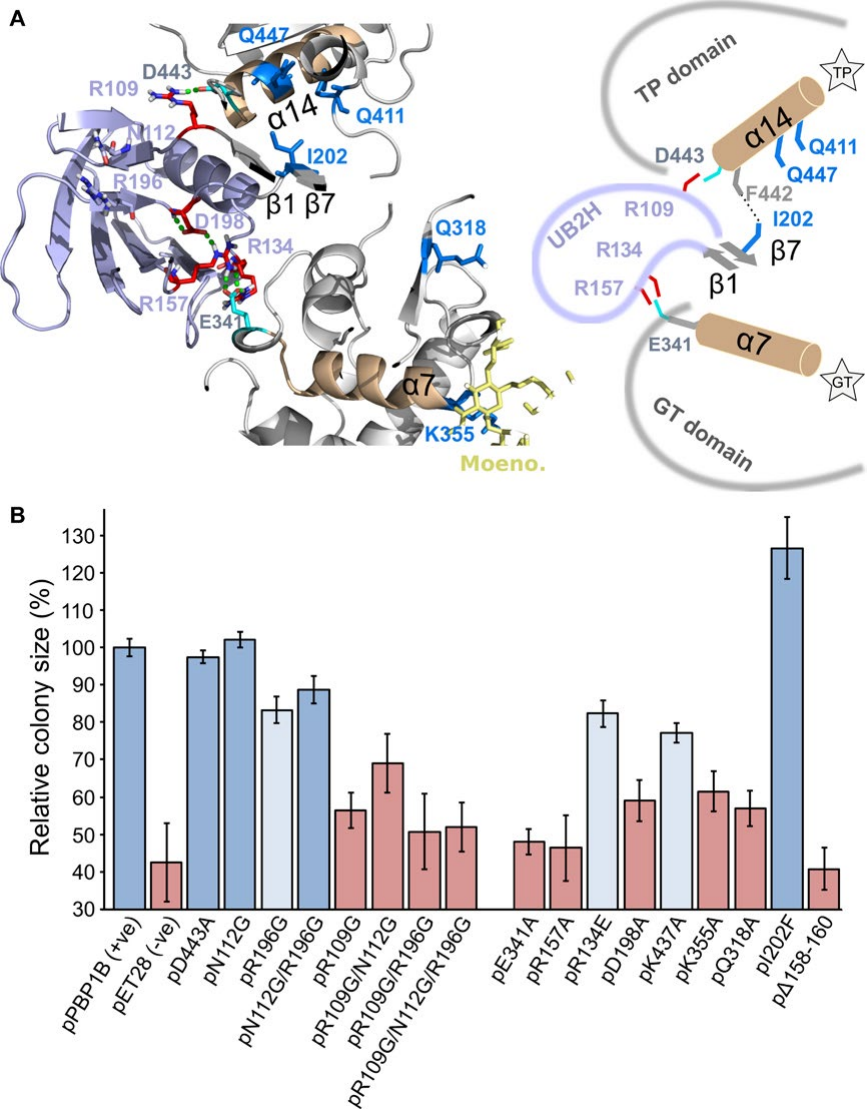
Perturbation of the putative activation pathways. A. H‐bond network connecting points of structural change in UB2H domain with the other domains of PBP1B (green dashed lines between residues) (PDB code 5FGZ). This network involves residues (coloured in red) mainly located in the N‐ and C‐terminal segments and in Loop 1 (residues 109‐114, 196‐200 and 157‐168, respectively) ultimately connecting the UB2H domain with residue D443 of the TPase domain, and residue E341 of the GTase domain. Residues coloured in blue represent the mutations of PBP1B that partially bypass the need of LpoB activation (Markovski et al., 2016). B. *In vivo* function of PBP1B versions as measured by cellular fitness under cefsulodin treatment (6 μg/mL). Cefsulodin primarily targets PBP1A, increasing the cell’s dependence on PBP1B. Colony size relative to the strain expressing WT PBP1B is used as proxy of cellular fitness; growth of the strain harbouring the empty vector represents basal growth (mean ± SD; *n* = 16). Each bar is coloured according to survival of the strain harbouring the specific PBP1B version once *mrcA* (encoding PBP1A) was deleted in three independent experiments. Blue, growth similar to WT; light blue, diminished growth compared to WT (≤ 50%); red, no growth. Expression of PBP1B versions from their pET28 vector in BW25113 Δ*mrcB* was not induced, relying on basal expression from a cryptic promoter (Egan et al., 2014). [Colour figure can be viewed at http://www.wileyonlinelibrary.com]

Based on these structural data, we identified two candidate pathways of conformational change through UB2H, one connected to the GTase domain via residues proximal to loop 1, and the other one connected to the TPase domain via the termini of UB2H (Fig. 1C and Table 1). We predicted that the substitution of charged polar residues R196 and D198 impacts the dynamics of Loop 1 and the positioning of R157 within it. R157 forms a H‐bond with E341, a residue proximal to the cap of α‐helix 7 in the GTase domain, and so reorientation of Loop 1 may in turn impact the orientation and/or movement of this helix (Fig. 2A). Helix 7 ends with K355 in the GTase domain active site cleft, and is proximal to Q318 in the glycan chain exit channel. Additionally we predicted that the local flexibility induced at the termini of UB2H by disruption of the β1′/β7′ β‐sheet may impact the orientation and/or movement of α‐helix 14 in the TPase domain, the capping residue of which (D443) forms a H‐bond with R109 in this region. Additionally, we investigated how the PBP1B mutant allele I202F derived from the genetic screen of Markovski *et al.* (2016) may achieve its LpoB bypass effect and found that this substitution may reorient local stacking of aromatic residues impacting the inter‐domain region of PBP1B proximal to both Loop 1 and the base of α‐helix 14, including residue K437. We constructed 17 PBP1B mutant alleles carrying substitutions in amino acids predicted to be part of each pathway or in the inter‐domain region, our reasoning for each substitution is summarised in Table 1.

**Table 1:** PBP1B activation signal perturbation versions

PBP1B version	Domain/position	Substitution rationale
R196G	UB2H/termini β‐sheet	Chemical shift perturbation (CSP) observed in presence of LpoB. Directly upstream of LpoB interacting residues E187, N188, R190 and Q191. Substitution with Gly expected to induce local structural flexibility.
D198A	UB2H	CSP observed in presence of LpoB, H‐bond with R157. Substitution with Ala will abolish H‐bond.
R134E	UB2H	H‐bonds with E341 and R157. Charge‐flip substitution expected to disrupt H‐bond and alter local structure/dynamics.
R157A	UB2H/loop 1	Connected to destabilised β‐sheet through H‐bonding with D198. H‐bond with E341. Substitution to Ala will disrupt this H‐bond.
E341A	GTase	Connection point between UB2H and GTase via H‐bond with R157. Proximal to cap of α‐helix 7. Substitution with Ala will disrupt the H‐bond to R157 and R134.
K355A	GTase/active site cleft	Residue at opposite end of α‐helix 7 to E341, proximal to essential catalytic E233 residue in active site cleft. We expected substitution of this charged residue with Ala to affect local structure/dynamics in the GTase catalytic site.
Q318A	GTase/glycan exit channel	Proposed to form the base of the GT donor binding cleft. Substitution to Ala to remove large polar side chain.
K437A	Inter‐domain region	Proximity with the extremity of UB2H Loop 1. We expected substitution of this charged residue with Ala may disrupt local structure dynamics.
I202F	Inter‐domain region	Version found by Markovski et al. which increases PBP1B activity and partially bypasses reliance on LpoB.
R109G	UB2H	CSP observed in presence of LpoB. H‐bond with D443. Substitution with Gly expected to induce local structural flexibility and abolish its H‐bond with D443.
N112G	UB2H/termini β‐sheet	CSP observed in presence of LpoB. Part of β1′‐strand. Substitution with Gly expected to induce local structural flexibility and disrupt the β1′‐strand.
D443A	TPase/α14	Caps α‐helix 14 in the TPase domain, H‐bond with R109 in UB2H. We expected substitution to disrupt the H‐bond with R109 and affect structural dynamics of α‐helix 14.

#### Blocking or mimicking the putative activation pathways

For the putative pathway to the GTase domain we sought to block the H‐bonding network by substitution of D198, R157 and E341 to Ala, and potential downstream effects of the reorientation of α‐helix 7 in the GTase domain by substituting K355 and Q318 to Ala. We substituted R196 in strand β7′ for Gly in an attempt to induce local flexibility and possibly mimic the activation signal. Additionally we substituted R134, a residue which also H‐bonds with E341, to Glu to assess the effect of a charge reversal on activity. For the putative pathway through the UB2H termini we sought to mimic the signal by substitution of R109 and N112 to Gly, attempting to destabilise the β‐sheet artificially inducing local flexibility, and to abolish H‐bonding of α‐helix 14 with this region by substituting D443 with Ala (Fig. 2A). R196G may also impact this pathway as it is situated proximal to this β‐sheet.

Seventeen PBP1B versions carrying substitutions were assessed for *in vivo* function by two genetic experiments. Throughout this work, the expression plasmids carrying *mrcB* alleles were sequenced to confirm the correct mutation. Strains lacking the native *mrcB* gene (encoding PBP1B) and expressing different PBP1B versions from an expression plasmid had their fitness measured in the presence of cefsulodin at concentrations below the MIC. Cefsulodin is a β‐lactam specific for PBP1A and PBP1B, but which targets mainly PBP1A function leaving the cells dependent on PBP1B for growth (Yousif et al., 1985; Denome et al., 1999; Curtis et al., 1979). The expression of each version was confirmed by western blot (Fig. S2A). The second experiment tested whether these same strains could survive after deletion of the gene encoding PBP1A (*mrcA*) (Fig. 2B). The two approaches gave consistent results (Fig. 2B).

Disruption of the GTase activation pathway resulted in a loss of PBP1B *in vivo* function, with substitution of R157 and E341 particularly detrimental to fitness. R196G and R134E had a minor effect on fitness, and gave ~ half the number of colonies post *mrcA* deletion relative to WT. Attempted mimicry of the TPase activation pathway yielded versions whose *in vivo* fitness was equivalent to WT with the exception of R109G, which had a dominant negative impact. Whenever R109G was present PBP1B was rendered non‐functional regardless of additional substitutions (Fig. 2B). Substitution of K437 to Ala impeded fitness, and survival of cells after deletion of *mrcA* was poor compared to WT. Cells expressing PBP1B with the I202F substitution were fitter than those expressing WT PBP1B at concentrations of cefsulodin below the MIC.

**Figure 3:**
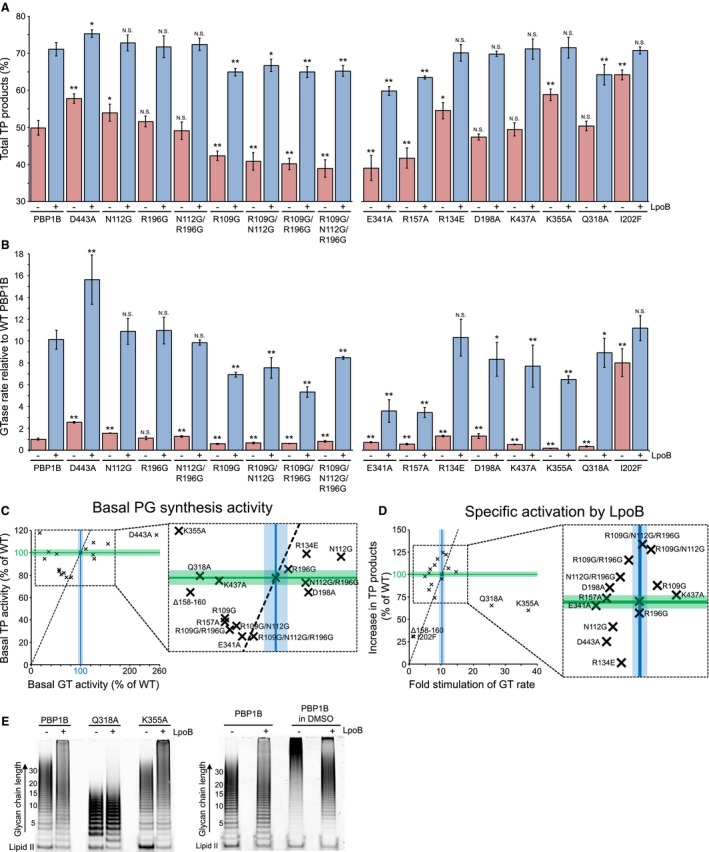
Perturbation of activation pathways impacts regulation of PBP1B. A. Total TPase activity of each PBP1B version. Data are the total products resulting from TPase domain activity (both peptide cross‐linking and minor carboxypeptidase) during *in vitro* PG synthesis using native [^14^C]‐lipid II substrate (mean ± SD, *n* = 3–4). The presence or absence of LpoB is indicated beneath appropriate bars with a + or ‐, respectively. Statistical significance was tested by paired T‐Test compared to the appropriate WT activity (basal version activity to basal WT, activation by LpoB to WT PBP1B + LpoB); N.S., not significantly different; ^*^, *P* ≤ 0.05; ^**^, *P* ≤ 0.005. B. GTase rates of each PBP1B version. Data are the rate of glycan polymerisation relative to WT PBP1B (mean ± SD, *n* = 3–4). The presence or absence of LpoB is indicated beneath appropriate bars with a + or ‐, respectively. Statistical significance was tested by paired T‐Test (as in panel a); N.S., not significantly different; ^*^, *P* ≤ 0.05; ^**^, *P* ≤ 0.005. C. Basal TPase and GTase activities of each PBP1B version correlation plot. The activity of each version was calculated relative to WT PBP1B activity as a percentage, relative GTase activity is plotted along the x‐axis against relative TPase activity on the y‐axis. WT PBP1B activity is displayed across the plot as coloured lines ± SD in corresponding lighter shade (TPase in Green, GTase in Blue). The dashed line represents the region on which data would fall if both activities have been altered to a similar magnitude. I202F activity is omitted from the plot as its inclusion skews the scale such that other points are illegible (basal GTase = 785% of WT, basal TPase = 128% of WT). D. Specific activation of each PBP1B version by LpoB. In this plot the fold GTase stimulation of each PBP1B version by LpoB is plotted on the x‐axis against the total increase in TP products produced by that version in the presence of LpoB relative to the increase caused by LpoB on WT PBP1B. WT PBP1B activation is displayed as coloured lines ± SD in corresponding lighter shade (TPase in Green, GTase in Blue). The dashed line represents the region on which data would fall if activation of both activities has been impacted to a similar magnitude. E. Glycan chain length assays of PBP1B versions. PBP1B versions, indicated above the corresponding lanes, were incubated with fluorescent lipid II substrate at the same conditions as for the PG synthesis and GTase assays with ampicillin to block cross‐linking. The presence of LpoB is indicated with a + above the appropriate lane, its absence with a ‐. After the reaction, resulting glycan chains were resolved by SDS‐PAGE and visualised by fluorescence imaging. The position at which the substrate runs is marked by ‘Lipid II’, glycan chain length is given in disaccharide units. [Colour figure can be viewed at http://www.wileyonlinelibrary.com]

Next, we purified 16 different PBP1B substitution versions for *in vitro* assay of the effect these changes had on GTase and TPase activity. All purified PBP1B versions were stable at 37°C for at least 1 h and bound the fluorescent β‐lactam Bocillin, indicating that their TPase domain was properly folded (Fig. S2B). We used two assays to measure basal activity and the stimulatory effect of LpoB on each version compared to WT. We used an end‐point PG synthesis assay with [^14^C]‐labelled lipid II substrate to quantify peptide cross‐linking and carboxypeptidase products as a percentage of the total PG product (we summarise these products of the TPase domain as ‘Total TPase products’). For reference throughout this section, WT PBP1B produced a PG with 49.9 ± 2% TPase products (Fig. 3A) which we refer to as its basal TPase activity. We also used a continuous fluorescence assay with dansylated lipid II, which cannot be used as TPase substrate, to measure GTase rate. In all results, these data are presented as the rate relative to WT PBP1B’s basal GTase activity, unless specified otherwise (Fig. 3B). From these data several classes of substitution were apparent: those that impact both activities positively, those impacting both negatively, and those that impact one or the other either positively or negatively. The impact of each substitution(s) on both basal activity and LpoB activation of the GTase and TPase domains relative to each other are shown in Fig. 3C and D. The *in vitro* activities observed are consistent with *in vivo* functionality (Fig. 2B).

#### Substitutions negatively affecting both GTase and TPase activities

As the GTase and TPase activities of class A PBPs are coupled (Bertsche et al., 2005; Born et al., 2006; Lupoli et al., 2014) we expected that some of the targeted substitutions we made (Table 1) to affect both of the two putative activation pathways. Consistent with its dominant negative effect on *in vivo* function, the presence of the R109G substitution decreased the basal GTase and TPase activities of PBP1B. TPase products were reduced to 42.3 ± 1.3%, and the GTase rate of PBP1B R109G was ~ half (0.55‐fold) of WT (Fig. 3A–C). Furthermore, while the R109G substitution did not prevent activation of the synthase by LpoB (Fig. 3D), the overall GTase and TPase activities did not reach WT levels.

A similar activity profile was observed for R157A and E341A, decreasing TPase products to 39.0 ± 3.4% and 41.5 ± 2.4%, respectively (Fig. 3A) and GTase rate to ~0.6 and 0.7‐fold of WT, respectively (Fig. 3B). As predicted, these substitutions also strongly impact on GTase activation by LpoB (Fig. 3B and D).

#### Substitutions positively affecting both activities

Another class of substitutions which affect both activities, in this case positively, include R134E, N112G and D443A. R134E, which was predicted to impact the GTase activation pathway (Fig. 2A), caused a minor increase in basal GTase (up ~1.3‐fold, Fig. 3B) and TPase (54.1 ± 1.8%, Fig. 3A) activities. Activation by LpoB increased the total activity to the same level as WT PBP1B suggesting the positive effect of the substitution could not be further enhanced (Fig. 3A–D). N112G and D443A substitutions were predicted to mimic the effect of LpoB binding on TPase activity. While these single substitutions were insufficient to fully mimic the activation signal both caused a significant increase in TPase activity compared to WT alone at 53.9 ± 2.3% with N112G and 57.8 ± 1% with D443A (Fig. 3A). N112G also caused a concomitant minor increase in basal GTase activity (up ~1.6‐fold, Fig. 3B) with no impact on overall activity in the presence of LpoB. D443A caused a more substantial increase in both basal GTase (up ~2.5‐fold) and in the activation by LpoB, giving a total rate at 15.6‐fold greater than WT PBP1B (Fig. 3B and C). Furthermore, D443A allowed the synthase to produce more TPase products in the presence of LpoB, up from 71.0 ± 1.8% to 75.3 ± 2.9% (Fig. 3A). Both the GTase and TPase data suggest that the D443A substitution enhances the stimulatory effect of LpoB, enabling even greater levels of activity than the WT, unlike for R134E and N112G alone.

I202F, a substitution allele of PBP1B selected as a suppressor of cefsulodin hypersensitivity in a ΔlpoB strain that can partially bypass the requirement for LpoB binding indeed had significantly increased basal activities. TPase products increased to 64.1 ± 1.3% (Fig. 3A) and GTase rate 7.9 ± 1.9‐fold compared to WT PBP1B (Fig. 3B). However neither was increased to the same level as WT PBP1B‐LpoB (at 71.0 ± 1.8% and 10.2 ± 0.9‐fold, respectively). In fact both activities were still increased by LpoB binding to levels equivalent to total WT PBP1B‐LpoB (Fig. 3A and B) suggesting that the I202F substitution indeed mimics the majority of the activation signal(s), and that LpoB binding cannot cause much further stimulatory change in this version, contrary to version D443A discussed above.

#### Combined Gly substitutions boost TPase activation by LpoB

Substitution of R196 to Gly, which we predicted may mimic GTase activation, had no effect on activity (Fig. 3A–D). While the R109G substitution had a negative impact on basal PBP1B activity and total activity in the presence of LpoB (Fig. 3A and B), stimulation by LpoB increased synthesis of TPase products similarly to the increase observed in WT PBP1B (Fig. 3D). However, when the R109G substitution is combined with the N112G and/or R196G substitutions the activation of the TPase activity of these versions by LpoB was increased compared to WT PBP1B (Fig. 3D). LpoB increased WT TPase activity by 21.1 ± 2.8%, while PBP1B versions R109G/N112G, R109G/R196G and R109G/N112G/R196G are activated by 25.8 ± 1.9%, 24.8 ± 1.0% and 26.3 ± 1.0%, respectively (Fig. 3D). This suggests that the presence of the multiple structure‐breaking Gly residues enhanced the effect LpoB binding has on the UB2H domain.

#### Substitutions affecting the GTase activity alone

D198A, a substitution we predicted to impact GTase activation, had no effect on basal TPase activity, or on its activation by LpoB (Fig. 3A). This substitution did however affect GTase activity, slightly increasing the basal rate (1.3‐fold) while impeding the activation by LpoB (Fig. 3B and D). Similarly, K437A, which is involved in a H‐bonding network with aromatic residues in the inter‐domain core of PBP1B and with the extremity of Loop 1, did not impact TPase activity (Fig. 3A and C) but affected GTase, inhibiting both basal activities (decreased to ~0.5‐fold of WT) and total activity in the presence of LpoB (Fig. 3B).

#### Downstream effects of the H‐bond connection between UB2H and the GTase domain

In seeking a rationale for why reorientation of the R157‐E341 H‐bond at the top of α‐helix 7 in the GTase domain (Fig. 2A) may activate catalysis we substituted residues K355 and Q318 to Ala. K355 is at the opposite end of this α‐helix 7 proximal to the essential catalytic residue E233. Q318 is connected indirectly to E341 through a H‐bonding network and forms part of the glycan chain exit channel of the domain. Substitution of either of these residues had a strong negative impact on basal GTase activity, K355A having weakest activity of all versions tested at 0.18 ± 0.03‐fold, and Q318A at 0.35 ± 0.06‐fold (Fig. 3B and C). Binding of LpoB to K355A and Q318A had the strongest stimulatory effect of all versions tested, significantly greater than the usual 10‐fold activation, at ~37‐ and 25‐fold, respectively (Fig. 3B and D). Despite this magnitude, activation was not sufficient to return total WT PBP1B‐LpoB levels of activity.

Unexpectedly, K355A also had a strong positive impact on basal TPase activity, increasing TPase products to 58.4 ± 2.1%. Similar to versions N112G, R134E and I202F with increased basal TPase activity the binding of LpoB only enhanced activity to the typical WT PBP1B‐LpoB level and no further. It is difficult to reconcile how such a distal substitution can have such an effect on TPase activation. This will be discussed below. Q318A had normal basal TPase activity, but impaired activation by LpoB synthesising 63.7 ± 1.9% TPase products. A possible explanation for this effect is the observation that this version made significantly shorter glycan chains than WT PBP1B (Fig. 3E), perhaps too short to allow high levels of TPase activity as the peptide substrate may not reach the active site as efficiently and/or frequently. Using a SDS‐PAGE based GTase assay, allowing visualisation of glycan chain length in the absence of TPase, in our conditions PBP1B produced glycan chains of ~20–25 disaccharide units in length on average. Contrary to previous reports we observed that LpoB induced PBP1B to produce glycan chains with a broader length distribution (Fig. 3E). It was reported that LpoB had the opposite effect, inducing PBP1B to produce shorter chains (Paradis‐Bleau et al., 2010). However, these data were obtained in the presence of 20% of the organic solvent DMSO and we replicate this solvent artefact (Fig. 3E). Q318A produced glycan chains with an average length of ~8 disaccharide units, and binding of LpoB had no effect on this length distribution (Fig. 3E). The defect in length is not caused by a general decrease in GTase activity as K355A, though much slower in rate than WT, shows the same length distribution as WT PBP1B with or without LpoB (Fig. 3E). This chain length defect, as well as the decrease in total TPase activity with LpoB, are likely the reasons the Q318A substitution cannot complement a loss of PBP1B in the cell (Fig. 2B).

### Structural dynamics is important for PBP1B activation

**Figure 4:**
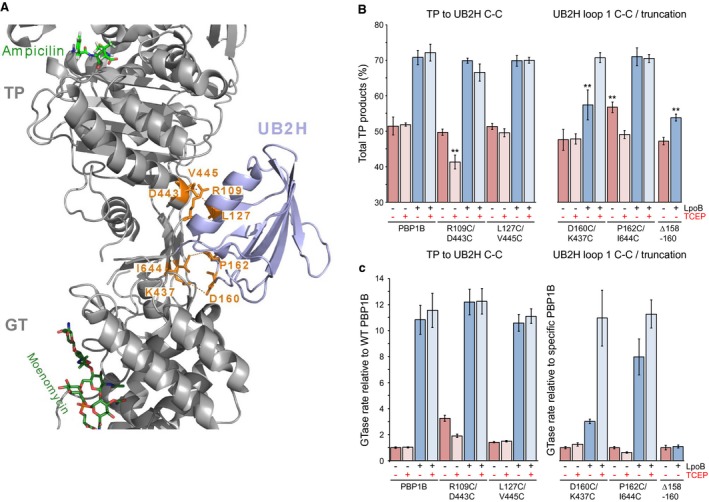
Disturbing structural dynamics impacts PBP1B regulation. A. Representation of short distances measured between the UB2H domain and the rest of PBP1B (PDB code 5FGZ). The residues coloured in orange are those replaced by Cys in pairs to form disulphide bridges between the UB2H domain and the TP or GT domain. B. Total TPase activity of PBP1B disulphide and truncation versions. Data are the total products resulting from TPase domain activity during *in vitro* PG synthesis using native [^14^C]‐lipid II substrate (mean ± SD, *n* = 3–4). The presence or absence of LpoB or TCEP is indicated beneath appropriate bars with a + or ‐, respectively. Statistical significance was tested by paired T‐Test compared to the appropriate WT activity (as in Fig. 3); ^*^, *P* = < 0.05; ^**^, *P* = < 0.005; no indication, no significant difference. C. GTase rates of each PBP1B disulphide and truncation versions. Data are the rate of glycan polymerisation relative to WT PBP1B or to the specific version as indicated to the left of each graph (mean ± SD, *n* = 3–4). The presence or absence of LpoB or TCEP is indicated beneath appropriate bars with a + or , respectively. [Colour figure can be viewed at http://www.wileyonlinelibrary.com]

Destabilisation of the terminal β‐sheet of UB2H induced by LpoB binding may increase structural dynamics in residues connected to this region via H‐bonding networks (Figs 1C and 2A) such as the Loop 1 residue R157, crucial for GTase activation signal transduction via H‐bonding with E341. We predicted that the dynamics of this loop and/or the connection between R157 and E341 may be required for signal transduction. Thus, we sought to alter the dynamics in two ways. Firstly we truncated Loop 1, removing residues 158–160 in an attempt to reduce overall flexibility. This version (PBP1B^Δ158–160^) was non‐functional in the cell (Fig. 2B). Secondly we introduced disulphide bridges between Loop 1 and the inter‐domain region of PBP1B with two double substitutions; D160C/K437C and P162C/I644C (Fig. 4A). Each of these three versions was purified and bound Bocillin (Fig. S2B). We detected no free thiol groups in either of the disulphide versions, or the WT PBP1B, by quantitation with DTNB reagent suggesting that the disulphide bridges are intact.

Truncation of Loop 1 strongly decreased basal PBP1B GTase activity to 0.27 ± 0.05‐fold that of WT (Fig. S3) without significantly impacting basal TPase activity (remaining similar to WT at 47.2 ± 1.0%). This truncation completely blocked the GTase activation signal from UB2H, with addition of LpoB having no effect on the polymerisation rate, which remained at 0.30 ± 0.07‐fold that of WT (Figs 4 and S3). Blocking the GTase activation signal in this manner also impacts the stimulation of TPase by LpoB giving only 53.8 ± 1.0% TPase products. Restricting motion of this loop by addition of a disulphide between its centre and the inter‐domain region of PBP1B (D160C/K437C) strongly inhibited activation of both GTase and TPase activities by LpoB (Fig. 4). This inhibition was completely reversed by addition of the disulphide reducing agent TCEP suggesting the effect was caused by the disulphide and not the substitutions themselves. Restricting the loop at its periphery, more distal to the R157‐E341 connection (P162C/I644C) also negatively impacted GTase activation but to a lesser degree. Again, this inhibition was reversed by reduction of the disulphide bond. Importantly, the addition of TCEP had no effect on WT PBP1B activity or stimulation by LpoB (Figs 4 and S3). Similarly to the K355A substitution, introduction of the disulphide between residues 162 and 644 had an unexpected positive effect on TPase activity. This will also be discussed below.

We hypothesised that disruption of the H‐bonding between the TPase domain and UB2H through R109 and D443 could increase TPase activity by allowing the whole domain more freedom of movement relative to the rest of the enzyme, consistent with a previously reported hypothesis based on the structure of some bifunctional PBPs (Lovering et al., 2008; 2012). We sought to test if this possible general increase in dynamics of the TPase domain was responsible by introducing disulphide bonds between the TP domain and UB2H through two double substitutions; R109C/D443C and L127C/V445C, the later residues not implicated in signal transduction, nor part of α‐helix 14 (Fig. 4A). These disulphides would, in theory, prevent release of the TPase domain if such a change happens. As above, these versions were purified, bound Bocillin (Fig. S2B) and had no detectable free thiol groups indicating the disulphides are in‐place. Introduction of a disulphide between residue 127 and 445 had no impact on PBP1B activation by LpoB, suggesting that overall dynamics of the TPase domain as a whole was not responsible for the increased activity. However, the connection between residues 109 and 443 appears to be important for basal PBP1B activity. When the connection was intact, the protein had normal TPase activity with increased basal GTase activity compared to WT by 3.2 ± 0.2‐fold (Fig. 4B and C). Upon reduction the GTase activity was decreased and basal TPase activity dropped to similar levels as those observed in R109G substitution versions (41.4 ± 2.0%) (Fig. 3A). Activation by LpoB was not significantly affected. This suggests that while the overall motion of the TPase domain relative to the rest of the protein is probably not a mechanism of stimulation by LpoB, the dynamics of α‐helix 14 within this domain is involved in controlling activity. Considered together with the effect of R109G on basal PBP1B activities these data suggest that this residue and its connections are crucial for maintaining an active conformation of the enzyme.

### Altering the active state of PBP1B affects LpoB binding

The affinity between some of the PBP1B versions and LpoB was measured by SPR (Table 2 and Fig. S4). LpoB bound to WT PBP1B with an apparent *K_D_* of ~0.5 μM and rapid on/off rate, consistent with our previous observations (Fig. S4) (Egan et al., 2014). The affinity between PBP1B I202F and LpoB was significantly higher, with an apparent *K_D_* value of 26 nM. Furthermore, the typically rapid on/off rate of the interaction between PBP1B and LpoB was altered in I202F, with a qualitatively decreased off‐rate (Fig. S4B). These data show that LpoB interacts more strongly with a more active PBP1B version. Consistent with this, other versions with increased basal activity, D443A and N112G, showed a minor but reproducible increase in affinities with apparent *K_D_* values of ~0.29 and 0.36 μM, respectively. PBP1B R109G, which had decreased basal activities, interacted with LpoB with a weaker affinity (*K_D_* of ~3 μM) suggesting that the converse is also true. Taken together these data suggest that LpoB binding to PBP1B is affected by the active state of the synthase. Exceptions to this observation are R157A and E341A, which both have decreased basal activities but retain affinities with LpoB similar to WT, or even increased in the case of E341A. We offer possible explanations in the discussion.

**Table 2:** Apparent dissociation constants of different PBP1B versions with LpoB and three CpoB constructs as determined by SPR

	Apparent dissociation constant, KD ± SD μM				
PBP1B version	LpoB	LpoB (reducing)	CpoB	CpoBTPR	CpoBCC
WT	0.491 ± 0.037		0.094 ± 0.030	4.9 ± 1.9	N.B.
R109G	3.09 ± 0.09		0.070 ± 0.003		
D443A	0.289 ± 0.017		0.095 ± 0.001		
N112G	0.356 ± 0.024		0.089 ± 0.002		
E341A	0.273 ± 0.024		0.077 ± 0.002		
R157A	0.505 ± 0.073	0.357 ± 0.040	0.147 ± 0.024		
I202F	0.026 ± 0.001	0.017 ± 0.001	> 2.0		
Δ158‐160	1.90 ± 0.29	1.70 ± 0.19	N.B.		
D160C/K437C	3.97 ± 0.51	2.04 ± 0.61			
P162C/I644C	0.213 ± 0.001	0.182 ± 0.035			

N.B., no binding detected. Reducing – dissociation measured in reducing conditions (2 mM TCEP in SPR running buffer).

### Mapping the CpoB interaction with PBP1B reveals the mechanism of inhibition

To understand why CpoB could have a negative effect on the stimulation of PBP1B TPase but not GTase activity by LpoB (Gray et al., 2015) we investigated the complex formation between CpoB and the UB2H domain. The X‐ray crystal structure of the trimeric N‐terminal domain of CpoB from *E. coli* was solved in 2010 (Krachler et al., 2010) but not for the C‐terminal domain. The C‐terminal domain structure was instead solved using the homologous domain from *Xanthomonas campestris*, and revealed an organisation as a TetratricoPeptide Repeat domain (TPR) (Krachler et al., 2010). Previously, we proposed that the C‐terminal TPR domain of CpoB interacts with PBP1B in *E. coli* (Gray et al., 2015). Here we have determined the structure of the C‐terminal TPR domain of CpoB from *E. coli* by NMR and found it adopts a fold similar to the *X. campestris* homologue (data deposited in BMRB and PDB, accession number 34256 and 6G5S, respectively). The domain is comprised of a series of seven helices that strongly and sequentially interact to form a TPR domain (Fig. S5). The structures of *E. coli* and *X. campestris* CpoB^TPR^ only differ in the N‐terminal helix orientation.

We validated our previous model of the CpoB^TPR^ (residues 139–263) interaction with PBP1B by SPR. Purified CpoB^TPR^ bound to PBP1B immobilised to a chip surface with an apparent *K_D_* of 4.9 ± 1.9 μM (Table 2, Fig. S4). This was a significant decrease in affinity compared to full length CpoB, which interacted with an apparent *K_D_* of 94 ± 30 nM. To explain this difference we assessed whether the N‐terminal ‘coiled‐coil’ domain is involved in the interaction, but found it to be dispensable. We then checked whether the inter‐domain linker region was involved, purifying a CpoB^TPR^ version with this linker (res. 110–263) but the affinity remained similar (Fig. S4). A perhaps key difference between CpoB and CpoB^TPR^ is that the later does not trimerise as effectively, with the majority existing in a monomeric state (Krachler et al., 2010).

**Figure 5:**
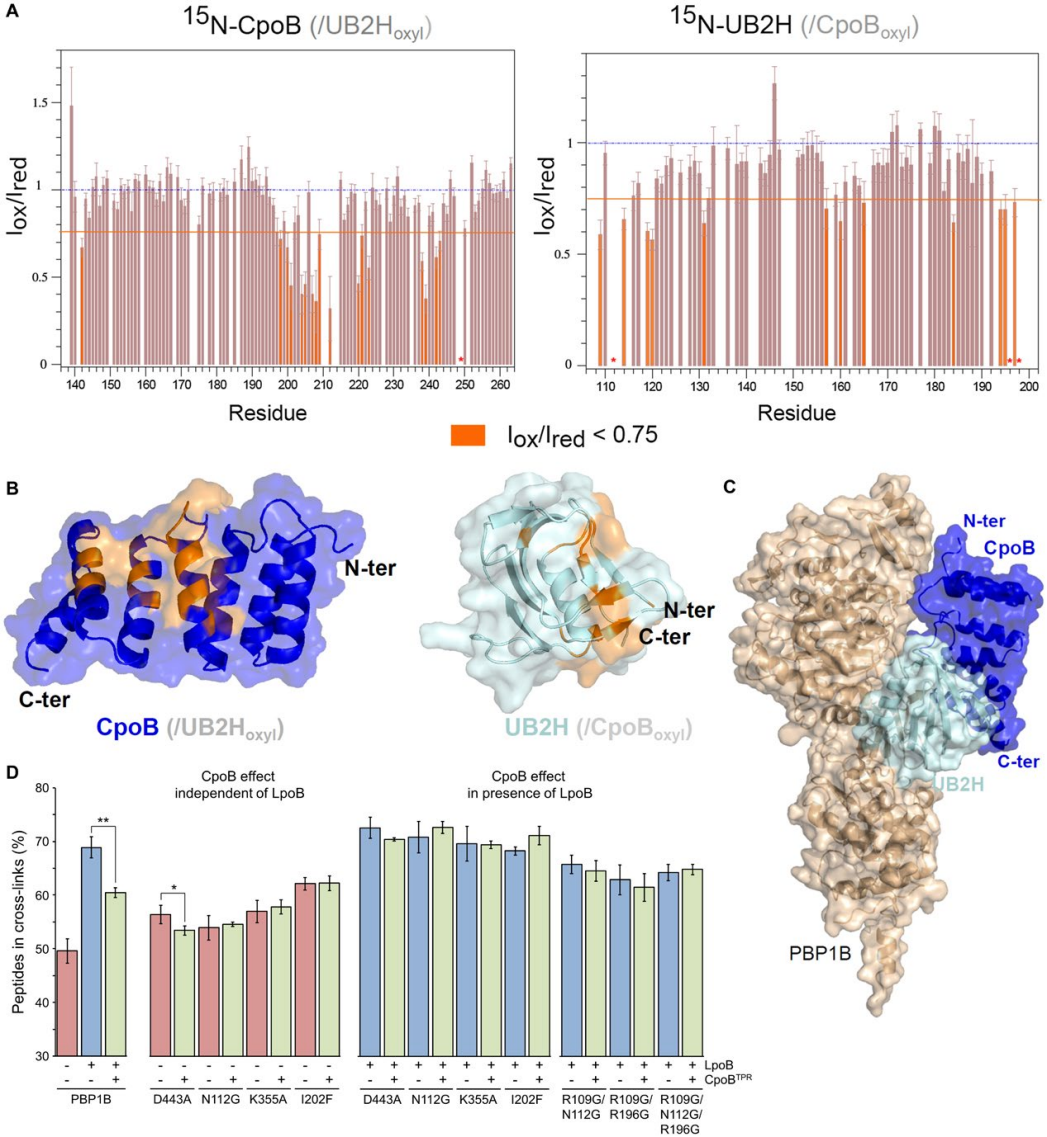
CpoB binds proximal to the TPase activation pathway in PBP1B. A. Paramagnetic Relaxation Enhancement (PRE) measured on NMR spectra of ^15^N‐CpoB and ^15^N‐UB2H after addition of Lys‐spin labelled UB2H and CpoB proteins, respectively. The quantification of PRE was obtained by measuring the intensity before and after reduction of the nitroxide group covalently linked to the epsilon amino group of the Lysine. Ratio that show a significant variation (< 0.75) are coloured in orange. Red stars represent resonances that completely disappear after addition of the paramagnetic protein. B. Residues with a PRE ratio < 0.75 are coloured in orange on the structure of CpoB (left) and UB2H (right). C. Data‐driven docking integrating PRE experimental data and previous cross‐linking data (Gray et al., 2015) was calculated from the structure of PBP1B (PDB 5FGZ) and the C‐terminus *E coli* CpoB domain lowest energy structure (PDB 6G5S). The lowest energy structure obtained with HADDOCK/CNS protocols is displayed with UB2H and CpoB domains coloured in pale blue and blue, respectively. D. Peptides in cross‐links produced by PBP1B versions in the presence of LpoB, CpoB, or both (mean ± SD, *n* = 3–4). Presence or absence of each regulator protein in reaction assays is indicted beneath each bar with a + or ‐, respectively. Statistical significance was tested by paired T‐Test compared to the appropriate activity (indicated by connecting line); ^*^, *P* ≤ 0.05; ^**^, *P* ≤ 0.005; no indication, no significant difference. [Colour figure can be viewed at http://www.wileyonlinelibrary.com]

To map the interaction surfaces between UB2H and CpoB, we have used Paramagnetic Relaxation Enhanced (PRE) NMR‐based detection (Clore and Iwahara, 2009; Hartl et al., 2012). In this approach, one binding partner is randomly spin labelled on lysine residues while the other partner is ^15^N isotopically enriched. Here we measured intensity ratios for [^15^N]‐CpoB and [^15^N]‐UB2H in the presence of the reciprocal spin labelled partner (Fig. 5A). Residues with an intensity ratio below 0.75 are coloured in orange on surface representations of the two proteins revealing regions of proximity (Fig. 5B). For CpoB, the proximity mainly localised in the α‐helix H4 (residues 198, 200, 201, 204, 205, 207), residues 208, 209, 212, α‐helix H5 (residues 220, 221, 223), and α‐helix H6 (residues 238, 239, 242, 243). For UB2H the affected residues were located in the N‐ and C‐terminal β‐sheet extremities (strands β1′ and β7′), which we have identified as playing a role in TPase activation by LpoB earlier in this work. Residues located in the Loop 1 (R157, D160, K165 and E166) were also affected. Additional proximities were observed in the loop formed by two anti‐parallel β‐sheets (residues 156–165 region). The PRE‐NMR data measured on UB2H and CpoB were used to calculate an updated model of the CpoB^TPR^‐PBP1B‐LpoB complex using the data‐driven HADDOCK molecular docking software by the same protocol as for our previously published model (Gray et al., 2015) (Fig. 5C).

Next we assessed whether CpoB^TPR^ modulates the activation of PBP1B by LpoB by counteracting the structural changes it induces in UB2H. We tested the effect of CpoB^TPR^ on the TPase activity of PBP1B versions which showed increased basal activity: D443A, N112G, K355A, I202F. The presence of CpoB^TPR^ decreased the peptides in cross‐links synthesised by PBP1B‐LpoB from 68.9 ± 2.0% to 60.5 ± 0.9%, a decrease in the proportion of stimulation of 43% (Fig. 5D). CpoB^TPR^ inhibited the increase in basal TPase activity of D443A by 43.4 ± 12.6%, a similar proportion to WT, suggesting this residue is involved in the dampening effect. D443A interacts with CpoB with the same affinity as WT PBP1B (apparent *K_D_* of 95 ± 1 nM) (Table 2). Conversely, N112G, K355A, and I202F are all immune to the effect of CpoB^TPR^, with its presence having no effect on their respective increases in basal TPase. Like D443A, N112G interacts with CpoB with a similar apparent *K_D_* to WT of 89 ± 2 nM, however I202F showed only weak binding to CpoB at the concentrations used. Given that CpoB^TPR^ has a 50‐fold lower *K_D_* than the full protein this particular observed immunity may be due to lack of sufficient binding instead of direct effect on the stimulatory structural changes.

We additionally tested the effect of CpoB^TPR^ on the above versions with LpoB, and on those versions which were stimulated by LpoB to a higher degree, R109G/N112G, R109G/R196G, R109G/N112G/R196G. In all cases the substitutions rendered PBP1B immune to the inhibitory effect in the presence of LpoB. R109G interacts with CpoB with a similar *K_D_* value of 70 ± 3 nM suggesting that this effect is not due to perturbed interaction in the multiple substitution versions. While the basal increase in D443A TPase was sensitive to CpoB^TPR^ modulation, the presence of LpoB overcomes this. This suggests CpoB^TPR^ is able to stabilise the effect D443A has on α‐helix 14 in isolation, but not in the presence of the additional allosteric changes induced by LpoB.

## Discussion

Despite the essentiality of class A PBPs in bacterial cell wall growth, their validation as effective antibiotic targets, and the identification of several protein interaction partners which exert regulatory effects, mechanistic insight into how these enzymes are controlled has remained unknown. In this work, to our knowledge we present the first molecular details towards understanding how two regulatory proteins LpoB and CpoB exert control on their cognate PG synthase PBP1B in *E. coli*. Our work is centred on the characterisation of stimulatory conformational changes induced in the regulatory domain of PBP1B, UB2H, by LpoB binding and how concurrent CpoB interaction with this domain selectively modulates these changes.

We found that binding of LpoB to UB2H caused structural changes, with a destabilisation of the anti‐parallel β‐sheet (strands β1′ and β7′) that connects the N‐ and C‐termini of the UB2H domain and an increase in dynamics of Loop 1 (Fig. 1). In this and our previous work on the UB2H‐LpoB interaction interface we observed that residues located upstream of the C‐terminus of UB2H, in an outward‐facing loop, are important for the interaction with LpoB (N188, R190, Q191) (Egan et al., 2014). We postulate that the interaction between those residues and LpoB can stretch the C‐terminal segment of UB2H and could be the reason for the drastic modification of the terminal β‐sheet in the isolated UB2H. In the full PBP1B structure, this destabilisation effect could cause local reorientation of UB2H relative to the two catalytic domains. Such a reorientation would have an effect on the H‐bond network that is formed between UB2H and the rest of the molecule. Based on this structural evidence, we have investigated a possible allosteric mechanism that could use the stability of the H‐bond network connecting the UB2H domain to the rest of PBP1B.

### PBP1B cycles between active and inactive conformations through structural dynamics

**Figure 6:**
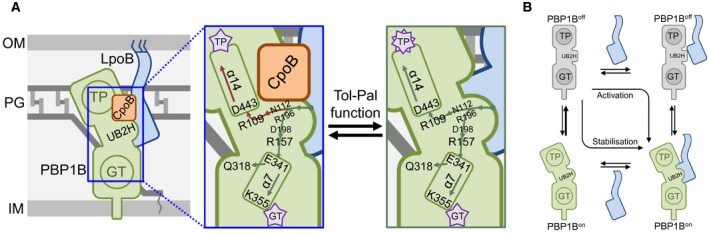
Regulation of PBP1B function. A. LpoB binding to UB2H activates PBP1B through two conformational rearrangement pathways. Binding destabilises the β‐sheet featuring N112 and R196 proximal to R109 at the base of TPase domain α‐helix 14 capped by D443. This change may reorient and/or increase motional dynamic of α‐helix 14, in turn impacting structure within the active site cleft promoting catalysis. LpoB binding also increased dynamic movement of UB2H Loop 1, from which R157 makes a H‐bond with the GTase domain via E341. Increased movement of this loop may reorient and/or increase movement of α‐helix 7 in the GTase domain, which connects to the active site cleft through K355, and to the glycan chain exit channel though Q318. This reorientation likely impacts the local structure around these residues, improving catalysis. Binding of CpoB, which may be the default state in the cell, is proximal to the TPase activation pathway and specifically dampens this activation signal by affecting the motion of α‐helix 14. This dampening is relieved in response to Tol‐Pal function (Gray et al., 2015). B. Cartoon representation of the two means of activation of PBP1B. PBP1B can exist in two states, inactive (off) or active (on). The synthase can be activated in two ways: (1) direct binding of LpoB induces the conformational changes shifting PBP1B to the active state (‘activation’), (2) the necessary conformational changes sporadically occur in PBP1B causing activation, and LpoB binding affinity is increased favouring binding and stabilisation of this active state (‘stabilisation’). Without LpoB to stabilise this state the frequency with which this sporadic activation occurs and/or the length of time PBP1B is active are not sufficient for effective PG synthesis in the cell. [Colour figure can beviewed at http://www.wileyonlinelibrary.com]

Our findings suggest that destabilisation of the terminal β‐sheet of UB2H constitutes part of the activation signal through two effects. A direct impact of this destabilisation is on α‐helix 14. One end of this helix is proximal to the TPase active‐site cleft, the other end interacts with residue R109 in the destabilised region of UB2H via D443. Upon destabilisation, α‐helix 14 is likely either slightly reoriented, more dynamic, or both, and this structural change improves catalysis. A secondary effect of the destabilisation of the terminal β‐sheet is on the dynamics of UB2H’s contact to the GTase domain via the R157‐E341 H‐bond proximal to Loop 1. Residue E341 in the GTase domain is directly connected to the cap of α‐helix 7. K355 at the opposite end of α‐helix 7 is proximal to the essential E233 catalytic residue in the GTase active cleft. King *et al*. noted that this proximity may be important in maintaining the crucial negatively charged form of E233 by electrostatic stabilisation (King et al., 2016). Indeed we found a critical role of K355 in GTase activity and found the version in which K355 is substituted for Ala to be extremely receptive to activation by LpoB (Fig. 3). Based on our data we postulate that the LpoB induced destabilisation of the UB2H β‐sheet increases dynamics of Loop 1, in‐turn increasing dynamic motion of α‐helix 7 which is favourable for catalysis. Together with the effect on α‐helix 14 this constitutes the allosteric activation pathways (Fig. 6A). Precisely how these changes boost the respective functions is unknown as NMR with the full length PBP1B‐LpoB complex is not feasible because of the molecular weight of >110 kDa and obtaining a co‐crystal of PBP1B in complex with LpoB is problematic, likely due to the highly dynamic nature of the interaction. The activation signal proposed here is consistent with previously identified versions of PBP1B, isolated through suppressor selection, which are able to partially bypass the need for activation by LpoB (Markovski et al., 2016). Two of these, I202F and Q411R, cluster in the inter‐domain region of PBP1B proximal to both α‐helix 14 and Loop 1. A third, Q447K, is part of α‐helix 14 (Fig. 2A). We currently have no insight into how a fourth suppressor versions isolated by Markovski *et al*., E313D, is exerting its bypass effect.

Our observation that the destabilisation effect driven by LpoB binding affects UB2H’s connection to the TPase and GTase domain explains how LpoB binding simultaneously stimulates both activities. It also offers one explanation for why many of the substitutions we made aiming to impact one or the other activation pathways in fact impacted both as they share the same root, the UB2H terminal β‐sheet. One residue however, had an opposite effect to our prediction and other residues nearby in the pathway. R109G caused basal TPase and GTase activity of PBP1B to decrease significantly. Furthermore, this substitution caused a decrease in the PBP1B‐LpoB affinity of ~6‐fold (Table 2). A simple explanation would be that its overall folding was disrupted, however it both bound Bocillin and interacted with CpoB with the same affinity as WT, suggesting that the protein is folded. Given that the strongly active I202F substitution increased LpoB binding affinity, and reduced the off‐rate of the binding, we concluded that while I202F exists predominantly in an active conformation R109G exists in an inactive one. These observations along with our data showing that structural dynamics are important for PBP1B activation led us to postulate that PBP1B is able to sporadically self‐activate through the same changes induced by LpoB binding, and that the R109G substitution causes the synthase to revert to the inactive state more rapidly by abnormal dynamics. The positive effect of a disulphide bridge between R109 and D443 supports this hypothesis (Fig. 4B). When intact, the active conformation is stabilised leading to the increase in basal GTase activity we observed, which is reversed once the connection is severed causing the synthase to be prone to reverting to the inactive state more rapidly similar to R109G. The ability to sporadically self‐activate would explain why the synthase is active in isolation *in vitro*. While the majority of the molecules are in an inactive or off state, a minority exist in the active state able to perform catalysis. This notion is consistent with previous observations (Markovski et al., 2016). The activity observed would be proportional to the longevity of this active state. It is likely that some of the other substitutions we made in probing the activation pathways also impacted the longevity of the active state explaining their effect on both activities, such as D443A and R134E, or had unexpected effect on one, such as the effect of K355A and the P162C/I644C disulphide on TPase activity.

### How does CpoB impair TPase activation?

By mapping the positions on UB2H to which CpoB^TPR^ interaction is proximal we find an explanation for its selective modulation of activation (Fig. 5). According to PRE NMR CpoB^TPR^ binding is on or near the upper portion of UB2H proximal to the TPase domain, including part of the destabilised β‐sheet, putting it in proximity to the TPase activation pathway through α‐helix 14. The increase in TPase activity caused by D443A substitution was dampened by CpoB^TPR^ to the same degree as WT PBP1B‐LpoB is, suggesting the inhibitory effect works to counter the conformational shift of α‐helix 14. Consistent with this, versions with multiple substitutions in this pathway that enhance β‐sheet destabilisation (R109G, N112G, R196G) are immune to the dampening effect of CpoB^TPR^. Together these interaction and activity data suggest that in the WT situation, binding of CpoB^TPR^ dampens the TPase activation pathway through stabilisation of the terminal β‐sheet region of UB2H (Fig. 6A). Whether the stabilisation is complete or not is unknown. If it were only partial it may explain why inhibition of the activation signal is not total. CpoB exists in 10× greater amounts than PBP1B in the cell (500 vs. 5000 copies) suggesting that the CpoB‐bound state is the default. Its inhibitory effect on PBP1B activation by LpoB is only relieved through the functioning of the Tol‐Pal system (Fig. 6A) (Gray et al., 2015). Of note is that PBP1B with a truncation of Loop 1 (Δ158‐160) showed no binding to CpoB with the protein concentrations tested (Table 2, Fig. S4). This result is in agreement with the chemical shift perturbations observed by NMR on UB2H (Fig. 5A) and with the proximity observed in the calculated model between the Loop 1 and CpoB^TPR^ (Fig. 5C). If indeed CpoB does also interact proximal to, or directly with, this loop its binding has no effect on the GTase activation signal in *E. coli* PBP1B, as we’ve previously reported (Gray et al., 2015). Perhaps *E. coli* CpoB is an exception, evolving to lose this ability, and versions from other species may exert a more total inhibition of their cognate class A PBP.

### Cellular context of PBP1B regulation

Taken together, our *in vivo* and *in vitro* data reveal a minimal level of PBP1B function required for growth (in the absence of the functionally redundant PBP1A). We observed that cells relying on PBP1B function need the synthase to have a GTase rate at least 8 to 9‐fold greater than our measured WT basal rate and the ability to produce at least 66% TPase products in the presence of LpoB. Any version, which has less activity than this, cannot support cell growth in the absence of PBP1A. This observed minimal requirement may explain why Markovski *et al.* (2016) could not delete both LpoB and PBP1A when cells were reliant on PBP1B I202F for growth, as the activity of this version in the absence of LpoB is below the observed threshold, with GTase activity 7.8 ± 1.2‐fold above WT and TPase activity at 64 ± 1.3%. So even though this version is much more active alone, it still requires LpoB for full activity.

While sporadic activation suffices for observation of PBP1B activity alone *in vitro*, this is insufficient in the absence of LpoB in the cell, as the minimal threshold is not reached. Thus, we propose that LpoB ensures PBP1B is functional in the cell in two ways: (1) binding of LpoB induces the required conformational changes directly, consistent with an induced fit model (Koshland, 1958; Zhang and Al‐Hashimi, 2009), as we’ve observed this effect on UB2H by NMR spectroscopy; (2) given that LpoB interacts stronger with more active conformations of PBP1B, when/if the synthase sporadically activates, LpoB binding is favoured which stabilises the active conformation, consistent with a tertiary capture model (Foote and Milstein, 1994; Zhang and Al‐Hashimi, 2009) (Fig. 6B). Without LpoB to induce and/or stabilise this state the frequency with which this sporadic activation occurs and/or the length of time PBP1B is active are not sufficient for the minimal PG synthesis threshold in the cell. This model is consistent with a previous report showing that limited PBP1B activity in the cell in the absence of LpoB is responsible for cell death (Markovski et al., 2016; Greene et al., 2018). R157A and E341A appear to be exceptions to this hypothesis as they have lower basal activities similar to R109G but retain similar affinity to LpoB as WT. Given that TPase activity requires ongoing GTase activity, such that catalysis is coupled, we postulate that the lower basal activity of these versions is not due to the substitutions affecting the longevity of the active state. Rather, it is because it blocks the GTase activation pathway. Even if the synthase sporadically self‐activates with the same frequency as WT, the activation signal cannot efficiently progress to the GTase domain to increase catalysis, in turn impacting the efficiency of the TPase.

There are numerous examples of class A PBPs across the bacterial kingdom, including representatives from both Gram‐positive and Gram‐negative phyla, which possess small non‐catalytic sequences or domains revealed either bioinformatically or when the protein’s structure was solved (Han et al., 2011; Typas et al., 2012; Jeong et al., 2013). In this work we have demonstrated how induced conformational changes in one such domain, *E. coli* PBP1B’s UB2H, control the activity of the synthase. While this work has focussed on the molecular details of this specific example, we expect that similar regulatory relationships exist wherever non‐catalytic domains/sequences are found, and that the specific activators simply remain to be discovered.

## Experimental procedures

### Chemicals and proteins

[^13^C]‐glucose was purchased from Cambridge Isotope Labs. The [^15^N]‐NH_4_Cl, and all other chemicals used, were purchased from Sigma‐Aldrich unless otherwise stated. Lipid II versions were prepared as previously described (Breukink et al., 2003; Bertsche et al., 2005). The following proteins and antibodies were prepared as previously described: LpoB (Egan et al., 2014), UB2H (Egan et al., 2014), CpoB (Gray et al., 2015), and anti‐PBP1B (Bertsche et al., 2006). CpoB^TPR^ and CpoB^CC^ versions were prepared by the same procedure as for CpoB. Cellosyl was provided by Hoechst AG, Frankfurt (Germany). *Bacillus cereus* β‐lactamase (569/H9) was purchased from Merck.

### Bacterial strains

Bacterial strains and plasmids used in this work are listed in Tables S2 and S3, respectively. Primers used in this work are listed in Table S4.

### Growth conditions

For growth assays, cells were grown aerobically at 30°C or 37°C in Lennox Luria‐Bertani (LB) medium (10 g/L tryptone, 5 g/L yeast extract, 5 g/L NaCl) or Miller LB (10 g/L tryptone, 5 g/L yeast extract, 10 g/L NaCl), or on solid versions of these supplemented with 1.5% (w/v) agar as indicated. Where appropriate, additional supplements were added: chloramphenicol (20 μg/mL), kanamycin (30 or 50 μg/mL), cefsulodin (6 μg/mL), sodium citrate (10 mM). For protein production, cells were grown aerobically at 30°C or 37°C in 1.5 L (unless otherwise stated) of Miller LB medium with 50 μg/mL kanamycin. Protein production was induced by addition of 1 mM IPTG once cells had reached OD_578_ 0.5–0.6 unless otherwise stated. Induction proceeded for at least 3 h at 30°C. For production of isotopically labelled proteins, kanamycin‐resistant BL21 (DE3) cells containing the plasmid of interest were grown in M9 minimal media with [^15^N]‐NH_4_Cl (1 g/L) for singly labelled or both [^15^N]‐NH_4_Cl (1 g/L) and [^13^C]‐glucose (2 g/L) for doubly labelled protein overexpression, as appropriate. First a 10 mL pre‐culture in Lennox LB was grown for few hours at 37°C to OD_600_ 1.0. This culture was back‐diluted (10‐fold dilution) in a second 100 mL pre‐culture in M9 containing appropriate isotopically labelled compound(s) and grown overnight at 37°C. 2 L of the M9 medium supplemented with vitamins (1 µg/L pyridoxine, 1 µg/L biotin; 1 µg/L D‐pantothenic acid hemicalcium salt, 1 µg/L folic acid, 1 µg/L choline chloride, 1 µg/L niacinamide, 0.1 µg/L riboflavin, and 5 µg/L thiamine) and salts (1 mM MgSO_4_, 0.1 mM CaCl_2_, 0.1 mM MnCl_2_, 50 µM ZnSO_4_, and 0.1 mM FeCl_3_) was inoculated to OD_600_ 0.2 and incubated at 37°C until the culture reached OD_600_ 1.0 at which point 1 mM IPTG was added to induce protein expression for 3 h at 30°C.

### Construction of plasmids

Expression vectors for oligo‐histidine tagged recombinant PBP1B^108–200^ (UB2H), CpoB^139‐263^ (CpoB^TPR^), CpoB^110‐263^ (CpoB^TPR+link^), and CpoB^27‐109^ (CpoB^CC^) with a thrombin cleavage site were created by insertion of the gene into pET28a at Nde I and Hind III restriction sites. Expression vectors for oligo‐histidine tagged PBP1B versions were created by mutagenesis of pDML924 encoding PBP1B^46‐844^ (Terrak et al., 1999). Mutagenesis was performed using a QuikChange lightning kit (Agilent technologies, USA) as per the manufacturer’s instructions.

### Construction of *E. coli* strains

To generate an antibiotic selection marker‐less *mrcB* deletion (Δ*mrcB*::FRT) the *kan*‐marked allele from the *E. coli* single gene knockout KEIO (Datsenko and Wanner, 2000; Baba et al., 2006) collection strain was transformed with plasmid pCP20 encoding a FLP recombinase, which excises the *kan* cassette by virtue of FRT sites flanking the *kan* allele leaving an FRT‐site scar in place (Cherepanov and Wackernagel, 1995). Δ*mrcB*::FRT was transformed with plasmids carrying PBP1B versions, including the WT and empty pET28a vector, to be used to assess growth fitness, and survival in the absence of both *mrcA* and *mrcB*. Expression of each PBP1B version was assessed by western blot detection of the protein from whole‐cell lysate of exponentially growing cells in Lennox LB at 30°C (Fig. S2A).

### Growth fitness assays

To generate *E. coli* strains in which growth is dependent on the specific plasmid‐borne PBP1B version P1 transduction was used to transfer Δ*mrcA*::*cat* into BW25113 Δ*mrcB*::FRT transformed with the pertinent plasmid (Thomason et al., 2007). Strains were grown overnight in 5 mL Lennox LB at 30°C. Cultures were pelleted, resuspended in 2 mL of 10 mM MgSO_4_, 5 mM CaCl_2_, pelleted again and finally resuspended in another 2 mL of 10 mM MgSO_4_, 5 mM CaCl_2_. 100 μL of cells were incubated at 30°C with 2 μL of P1 phage derived from BW38029 Δ*mrcA*::*cat,* after which 1 mL of Lennox LB with 30 μg/mL kanamycin and 10 mM sodium citrate was added. Cells were incubated at 30°C for 1 h with shaking before plating on Lennox LB with 30 μg/mL kanamycin, 20 μg/mL chloramphenicol and 10 mM sodium citrate. Plates were incubated at 30°C until visible colony growth. Colonies were screened by PCR to check for the absence of both *mrcA* and *mrcB* from the chromosome.

For quantitation of growth fitness of strains expressing each PBP1B version relative to cells expressing WT, PBP1B Δ*mrcB*::FRT transformed with the pertinent plasmids were replica pinned on Lennox LB agar with 6 μg/mL cefsulodin in two independent arrays. Each strain was pinned in quadruplicate in each array. Plates were incubated at 37°C for 12 h and imaged. Colony size was quantified using the image analysis software Iris (Kritikos et al., 2017).

### Protein purification

*E. coli* BL21(DE3) transformed with the appropriate plasmid was used for all protein production in this work. As indicated above UB2H, LpoB, CpoB (and its subdomains) were purified as previously described (Egan et al., 2014; Gray et al., 2015). In brief cells harvested by centrifugation were resuspended in 25 mM Tris/HCl, 500 mM NaCl, 20 mM imidazole buffer at pH 7.5, in the presence 10 mg lysozyme and RNase/DNase. Cells were disrupted by sonication and the cell debris were removed at 46,000×*g* for 45 min at 4°C. The supernatant was loaded on a 10 mL HisTrap chelating column (GE Healthcare, USA) and the protein was eluted using a 40 to 400 mM imidazole concentration gradient. The protein was further purified in 10 mM Tris/HCl, 200 mM NaCl buffer at pH 7.5 using a Superdex 200 size exclusion chromatography column (GE Healthcare, USA). The sample was concentrated for NMR experiments, for which the histidine‐tag was conserved. The procedure for PBP1B purification was modified from Bertsche *et al.* (2006) to improve efficiency and ensure consistency between preps. Protein production was performed as described above in ‘growth conditions’ at 30°C throughout. Cells harvested by centrifugation were resuspended in 80 mL of 25 mM Tris/HCl, 500 mM NaCl, 1 mM EGTA, 10% glycerol, pH 7.5 to which 1 in 1000 protease inhibitor cocktail (Sigma‐Aldridge) and 100 μM phenylmethanesulfonyl fluoride (PMSF) was added. The resuspension was frozen at 80°C until required (< 3 months), at which time it was rapidly thawed and cells disrupted by sonication. The membrane fraction was pelleted by centrifugation at 130,000 × *g* for 1 h at 4°C and resuspended in 25 mM Tris/HCl, 5 mM MgCl_2_, 1 M NaCl, 20% glycerol, 2% Triton X‐100, pH 7.5 with protease inhibitor cocktail and PMSF added as before. Extracted membranes were again centrifuged at 130,000 × *g* for 1 h at 4°C to remove remaining insoluble debris before 1:1 dilution with 25 mM Tris/HCl, 5 mM MgCl_2_, 1 M NaCl, 40 mM imidazole, 20% glycerol, pH 7.5 and application to an equilibrated 5 mL HisTrap column attached to an ÄKTA Prime^+^ system (GE Healthcare, USA) with fraction collection. Once the sample had been fully applied the column was washed with 40 mL of 25 mM Tris/HCl, 5 mM MgCl_2_, 1 M NaCl, 40 mM imidazole, 20% glycerol, 0.2% Triton X‐100, pH 7.5. Bound His_6_‐PBP1B was eluted stepwise with 25 mM Tris/HCl, 5 mM MgCl_2_, 1 M NaCl, 400 mM imidazole, 20% glycerol, 0.2% Triton X‐100, pH 7.5. His_6_‐PBP1B containing fractions were pooled in regenerated cellulose dialysis membrane with a molecular weight cut‐off of 6–8 kDa (Spectrum Labs, USA) and treated with 50 U/mL thrombin (Novagen, Merck, USA) for 20 h at 4°C during dialysis against 25 mM Tris/HCl, 5 mM MgCl_2_, 1 M NaCl, 20% glycerol, pH 7.5. Protein was then dialysed in preparation for ion exchange chromatography, first against 20 mM NaOAc, 1 M NaCl, 10% glycerol, pH 5.0; then against the same buffer with 300 mM NaCl; and finally against the same with 100 mM NaCl immediately prior to application to an equilibrated 1 mL HiTrap SP HP column attached to an ÄKTA Prime^+^ system (GE Healthcare, USA) with fraction collection. The column was equilibrated in 20 mM NaOAc, 100 mM NaCl, 10% glycerol, 0.02% NaN_3_, 0.2% Reduced Triton X‐100, pH 5.0 (buffer A). Once the sample had been applied the column was washed with 5 mL buffer A before elution of bound protein by gradient. The gradient was from 0 to 100% buffer B (as A, with 2 M NaCl) over 14 mL. PBP1B elution peaks at ~75% B. To ensure consistency between all PBP1B version preparations, particularly that the detergent concentration remained constant, 2 L of ion exchange buffers A and B were prepared and used for all preparations. PBP1B containing fractions were pooled and dialysed in 3 mL volume dialysis cassettes (D‐Tube maxi, molecular weight cut‐off 6–8 kDa, Merck, USA) against 20 mM NaOAc, 500 mM NaCl, 20% glycerol, pH 5.0. As Triton X‐100 micelles do not pass through the dialysis membrane the final buffer conditions are 20 mM NaOAc, 500 mM NaCl, 20% glycerol, 0.2% Reduced Triton X‐100, pH 5.0.

### Paramagnetic spin labelling of proteins

We use OXYL‐1‐NHS (1‐oxyl‐2,2,5,5‐tetramethylpyrolline‐3‐carboxylate‐N‐hydroxysuccimide ester; Toronto Research Chemicals Inc.) in order to randomly paramagnetically label the є‐amino groups of lysines in CpoB or UB2H as previously described with modifications (Lawrence et al., 1980; Hartl et al., 2012). A 178 mM stock solution of OXYL‐1‐NHS was prepared in DMSO and stored under argon at 20°C. Protein was labelled in a buffer of 10 mM Na_2_CO_3_, pH 9.2 at 20 µM. OXYL‐1‐NHS was added at 120 μM and the mixture was incubated at room temperature for 1 h and at 4°C for an additional 4 h. Finally, the excess of OXYL‐1‐NHS was removed from the reaction mixture by using a 10 kDa cut‐off concentrator and washing the sample with at least 20 volumes of 10 mM Tris/HCl, 200 mM NaCl, buffer at pH 7.5 and the protein concentration was adjusted to 100 µM in this buffer.

### NMR spectroscopy

For structure determination, NMR spectra were recorded at 20°C on a 0.5 mM [^13^C,^15^N]‐UB2H sample in 10 mM Tris/HCl, 200 mM NaCl buffer at pH 7.5 containing 10% D_2_O, or a 2 mM [^13^C,^15^N]‐UB2H sample with 1.2 molar ratio of unlabelled LpoB in the same buffer, or a 2 mM [^13^C,^15^N]‐CpoB^TPR^ sample in the same buffer. For each sample assignment of ^1^H, ^13^C and ^15^N backbone and side‐chain resonances was performed using a set of 3D heteronuclear experiments including BEST‐TROSY‐HNCACB, BEST‐TROSY‐HN(CO)CACB, HNCO, HN(CA)CO, hCCH‐TOCSY and HcCH‐TOCSY. These datasets were collected on Bruker Avance spectrometers operating at 700 or 800 MHz 1H‐frequency equipped with triple‐resonance cryogenic probes. 3D ^15^N‐NOESY‐HSQC, 3D aliphatic and aromatic ^13^C‐NOESY‐HSQC experiments were acquired with mixing times of 150 ms on a Bruker Avance US2 spectrometer equipped with a triple‐resonance cryogenic probe to obtain structural restraints (see below). Data were processed using Bruker software and analysed in CcpNmr (Vranken et al., 2005).

### Extraction of structural restraints and structure calculation

Distance restraints from 3D ^15^N‐NOESY‐HSQC, as well as from 3D aliphatic and aromatic ^13^C‐NOESY‐HSQC experiments, were obtained after automatic peak picking and assignment performed by UNIO'10 version 2.0.2 (Guerry and Herrmann, 2012). TALOS+ was used to calculate the chemical shift index (using HN, N, Cα, Cβ and CO chemical shifts extracted from CcpNmr), to calculate the S^2^ parameters of the backbone amide and to determine dihedral angle restraints from chemical shifts (Shen et al., 2009). The structures of UB2H alone, UB2H in complex with LpoB, and CpoB^TPR^ were calculated with Aria 2.3.1 using 100 structures for each of the 8 iterations, with the exception of the last cycle where 750 structures were calculated (Rieping et al., 2007). The 20 lowest energy structures from the last iteration underwent explicit water refinement in the NMR module of the Crystallography and NMR System (Brünger et al., 1998). Structures were visualised with Pymol Molecular Graphics System, Version 1.5.0.4 (Schrödinger, LLC) for data analysis and figure preparation.

### *In vitro* protein interaction and activity assays

The interactions among UB2H, LpoB and CpoB were evaluated by NMR in solution at 850 MHz, by following the intensity changes of the cross peaks on the 2D HN‐HSQC spectra recorded at 20°C. All protein interaction assays were performed by using at least one ^15^N‐labelled protein sample, with the other protein partners non‐labelled or randomly spin labelled on the є‐amino groups of lysine residues. The molar ratio of labelled *versus* unlabelled protein was 1:2 and a 100 µM concentration in the mixture was used for the ^15^N‐labelled protein. The paramagnetic effect on ^15^N‐UB2H and ^15^N‐CpoB was reduced by addition of sodium ascorbate at a concentration of 1.2 and 1.6 mM, respectively. The quantification of PRE (paramagnetic relaxation enhancement) for each resonance was carried out by the ratio of signal intensity in the oxidised and reduced states, before and after addition of sodium ascorbate, respectively:

$$I_{\text{PRE}}=\;I_{\text{ox}}\text{/}\;I_{\text{red}}$$

The interaction affinities of LpoB and CpoB versions of PBP1B were measured by Surface Plasmon Resonance assays as described previously with minor changes (Egan et al., 2014; Gray et al., 2015). The concentration of LpoB injected ranged from 19.5 nM to 10 μM. The concentration of CpoB versions injected ranged from either 3.9 nM to 2 μM or from 78 nM to 40 μM. To assay binding in reducing conditions 2 mM TCEP was included in the SPR running buffer, and PBP1B versions immobilised to the chip surface were incubated in this buffer for 30 min before the initiation of binding assays. The dissociation constant (*K_D_*) was calculated by non‐linear regression using SigmaPlot 11.5 software (Systat software Inc., USA) for at least three independent experiments.

Bocillin binding of the purified PBP1B versions was assessed by incubating 1 μg of each version in 20 μL of 20 mM HEPES/NaOH pH 7.5, 5 mM MgCl_2_, 150 mM NaCl, 0.05% Triton X‐100 with or without 1 mM ampicillin at 37°C for 30 min. After which 50 ng of BOCILLIN^TM^ FL (Bocillin; Invitrogen, USA) was added before further incubation at 37°C for 30 min. 15 μL of each sample was resolved by SDS‐PAGE, the gel then imaged by Typhoon scanner 9400 with excitation and emission filters of 488 and 520 nm, respectively. The same gel was then stained using Coomassie Brilliant‐Blue.

All activity assays were performed with 0.5 μM PBP1B (or versions there‐of) with 2 μM LpoB, 50 μM CpoB^TPR^ or both as indicated. Continuous fluorescence GTase assays were performed as described previously, in a buffer of 50 mM HEPES/NaOH pH 7.5, 25 mM MgCl2, 150 mM NaCl, 0.05% Triton X‐100 at 30°C (Egan and Vollmer, 2016). Measurement of TPase activity using radiolabelled lipid II substrate was performed as described by Bertsche *et al*. in a buffer of 20 mM HEPES/NaOH pH 7.5, 5 mM MgCl_2_, 150 mM NaCl, 0.05% Triton X‐100 at 37°C (Bertsche et al., 2005). In activity assays (either of the above) performed at reducing conditions, 10 mM TCEP (pH adjusted to 7.5 with NaOH) was added and proteins were incubated for 20 min at RT before proceeding. Measurement of glycan polymer lengths was performed largely as described previously with modifications to reaction conditions and the labelling of lipid II (Barrett et al., 2007; van’t Veer et al., 2016). Reactions were performed in a buffer of 20 mM HEPES/NaOH pH 7.5, 5 mM MgCl_2_, 150 mM NaCl, 1 mM ampicillin, 0.05% Triton X‐100 at 37°C. 20% DMSO was included where indicated. Substrate was prepared as follows: 5 μM ATTO^550^‐labelled lipid II (analogous to Dansyl‐lipid II used for the continuous GTase assay) was mixed with 25 μM lipid II in a solvent of 1:1 chloroform methanol. The mixture was dried, lipid II was then dissolved in 0.1% Triton X‐100. Reactions were initiated by addition of the remaining components to the substrate solution to a final volume of 50 μL. After 1 h incubation at 37°C 15 μL was removed and concentrated to 1 μL or dryness in a Scanvac vacuum concentrator (Labogene, Denmark), for reactions containing DMSO the centrifuge was set to 37°C to accelerate concentration. Once dried, the resulting material was dissolved in 4 μL sample buffer and resolved on Tris‐Tricene SDS‐PAGE gel. Once resolved the gel was imaged by Typhoon scanner 9400, with excitation and emission filters at 532 and 580 nm, respectively.

### Quantitation of free thiol groups in protein

Duplicate samples of 2 μM PBP1B version or L‐Cys standards from 2 to 128 μM in 50 mM HEPES/NaOH, 5 mM MgCl_2_, 150 mM NaCl and 0.05% Reduced Triton X‐100, pH 7.5 were incubated in a 96‐well plate at RT for 5 min prior to addition of 6 M GdnHCl (100 μL final sample volume) to denature protein. Samples were further incubated for 10 min at RT prior to addition of 100 μL DTNB reaction solution (0.4 mM DTNB, 0.1 M Na_2_HPO_4_, 1 mM EDTA, 6 M GdnHCl, pH 8.0). The reaction was incubated for 15 min at RT before measurement of absorbance at 405 nm in a Tecan Infinite F50 microplate reader (Tecan, Switzerland).

### Data‐driven structural docking models

The docking models of CpoB/PBP1B were built using HADDOCK2.1 data‐driven docking protocols (Dominguez et al., 2003) and CNS1.2 (Brünger et al., 1998) for the structure calculations. The model was calculated with the same protocol as described previously (Gray et al., 2015) but using the *E. coli* C‐terminal domain of CpoB determined by NMR (PDB 6G5S). In addition to the previous restraints defined by *in vivo* cross‐linking between CpoB and PBP1B, quantification of PRE was used to define supplementary intermolecular restraints. Residues with a PRE ratio of 0.75 (Fig. 5A) were added as active restraints (i.e. residues 109, 114, 119, 120, 131,132, 157, 160, 165, 184, 194, 195 and 197 for UB2H and residues 198, 200, 201, 204, 205, 207, 208, 209, 212, 220, 221, 223, 238, 239, 242 and 243 for CpoB^TPR^).

### Accession numbers

Coordinates of 20 structures and chemical shifts of UB2H with/without LpoB have been deposited in the Protein Data Bank and the BioMagResBank under the accession codes 6G5R/6FZK and 34255/34246, respectively. Coordinates of 20 structures and chemical shifts of CpoB^TPR^ have been deposited in the Protein Data Bank and the BioMagResBank under the accession codes 6G5S and 34256, respectively.

## Author contributions

AJFE and RM‐M; conception and design, acquisition of data, analysis and interpretation of data, drafting and revising the article. IA, CMB, MB; acquisition of data, analysis and interpretation of data, revising the article. EB, preparation of key reagents, revising the article. WV and J‐PS; conception and design, analysis and interpretation of data, drafting and revising the article.
