# Supplementary material for: Induced conformational changes activate the peptidoglycan synthase PBP1B
Source: Mol Microbiol. 2018 Oct 25;110(3):335–56. doi: 10.1111/mmi.14082 (PMC6220978; doi:10.1111/mmi.14082)
Supplement: Supplementary file 1 [file MMI-110-335-s001.pdf]

Supporting information

Induced conformational changes activate the peptidoglycan synthase PBP1B

Alexander J. F. Egan[1,†], Roberto Maya-Martinez[2, †], Isabel Ayala[2], Catherine M. Bougault[2], Manuel Banzhaf[3,4], Eefjan Breukink[5], Waldemar Vollmer[1]* and Jean-Pierre Simorre[2]*

[1] The Centre for Bacterial Cell Biology, Institute for Cell and Molecular Biosciences, Newcastle University, Richardson Road, Newcastle upon Tyne, NE2 4AX, United Kingdom.
[2] Univ. Grenoble Alpes, CNRS, CEA, Institut de Biologie Structurale (IBS), 71 avenue des Martyrs, 38000 Grenoble, France.
[3] European Molecular Biology Laboratory Heidelberg, Genome Biology Unit, Meyerhofstraße 1, 69117 Heidelberg, Germany.
[4] Institute of Microbiology & Infection and School of Biosciences, University of Birmingham, Edgbaston, Birmingham, UK. B15 2TT.
[5] Bijvoet Center for Biomolecular Research, Department of Biochemistry of Membranes, University of Utrecht, Padualaan 8, 3584 CH Utrecht, The Netherlands.

[†] Authors contributed equally to the work.

*Correspondence:
Jean-Pierre Simorre: Institut de Biologie Structurale, 71 avenue des Martyrs, CS10090, 38044 Grenoble Cedex 9, France Email: jean-pierre.simorre@ibs.fr; phone: +33 (0) 457 428 555; fax: +33 (0) 476 501 890.

Waldemar Vollmer: The Centre for Bacterial Cell Biology, Newcastle University, Richardson Road, Newcastle upon Tyne, NE2 4AX, United Kingdom. Email: w.vollmer@ncl.ac.uk; phone: +44 (0) 191 208 3216; fax: +44 (0) 191 208 3205.

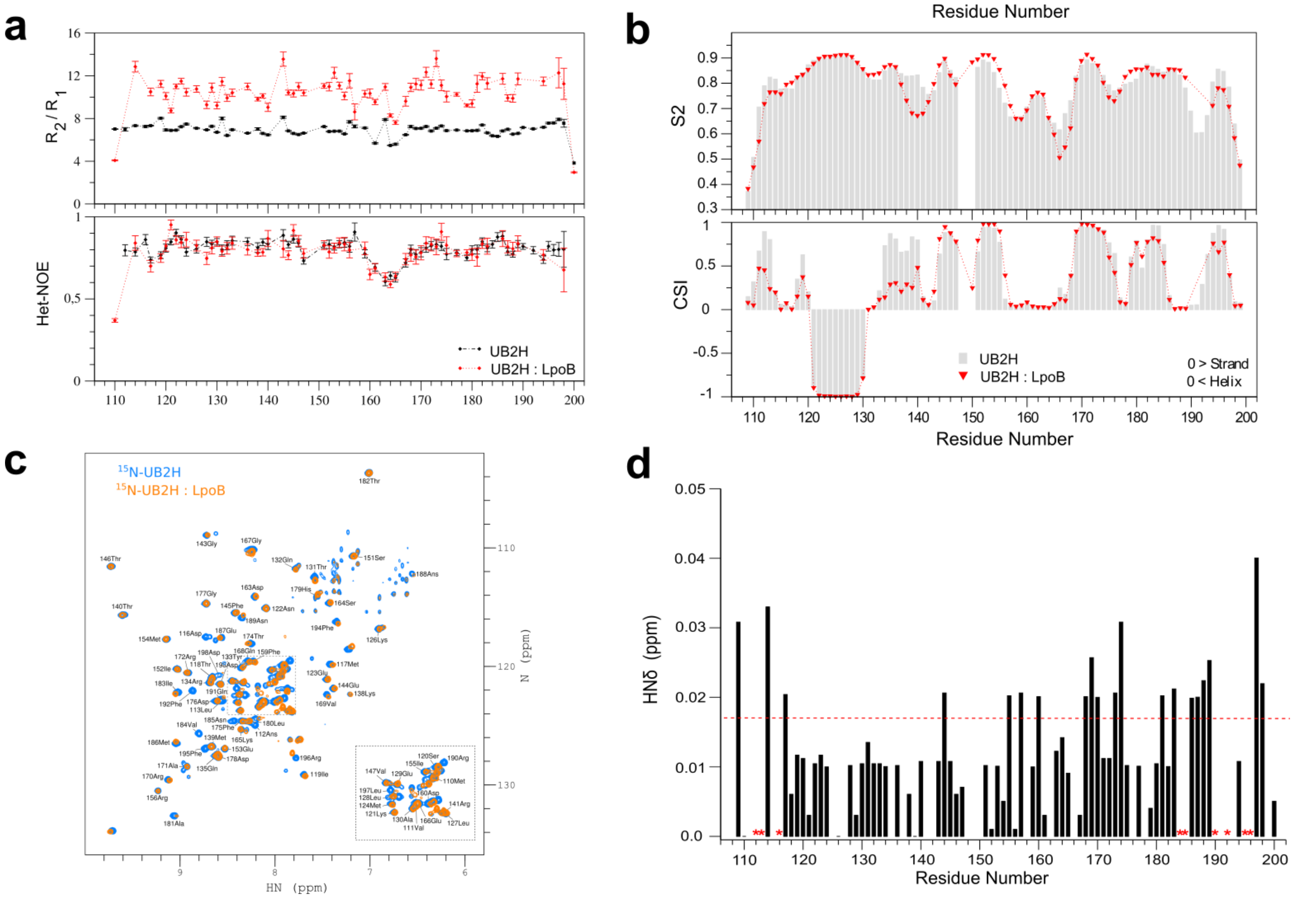

Supporting Figure 1: Comparison of NMR relaxation parameters and chemical shifts in UB2H and UB2H:LpoB

A, R_2/R_1 ratios and heteronuclear {^{1}H}-^{15}N NOE (Het-NOE) values measured at 20 °C, pH 7.5 and 700 MHz for isolated ^{15}N-labelled UB2H domain before (black) and after addition (red) of unlabelled LpoB at a ratio of 1:1.2.

B, Chemical shift index (CSI) and derived model-free order parameters (S^2) calculated from TALOS+ using H_N, N, Cα, Cβ, and CO chemical shifts measured for the free UB2H domain (grey bars) and the UB2H:LpoB complex (red triangles). CSI values identify secondary structure elements (CSI of 1 for β-strand, -1 for α-helix and 0 for random structure). S^2 order parameter varies from 1 to 0 with zero corresponding to completely disordered regions and values below 0.5 emphasizing highly flexible regions.

C, 2D-[^{1}H,^{15}N]-BEST-TROSY experiments recorded at 20°C and pH 7.5 for the ^{15}N-labelled UB2H domain before (blue) and after (orange) addition of unlabelled LpoB at a ratio of 1:1.2.

D, Chemical shift differences calculated from the data displayed in panel (C) as the weighted-average distance between the resonance position of the free form of UB2H and its equivalent position in the the UB2H:LpoB complex for each residue. Red horizontal dotted line represents the value of 2 standard deviations calculated all of the data. Red stars correspond to a residue for which the UB2H resonance disappears after addition of LpoB.

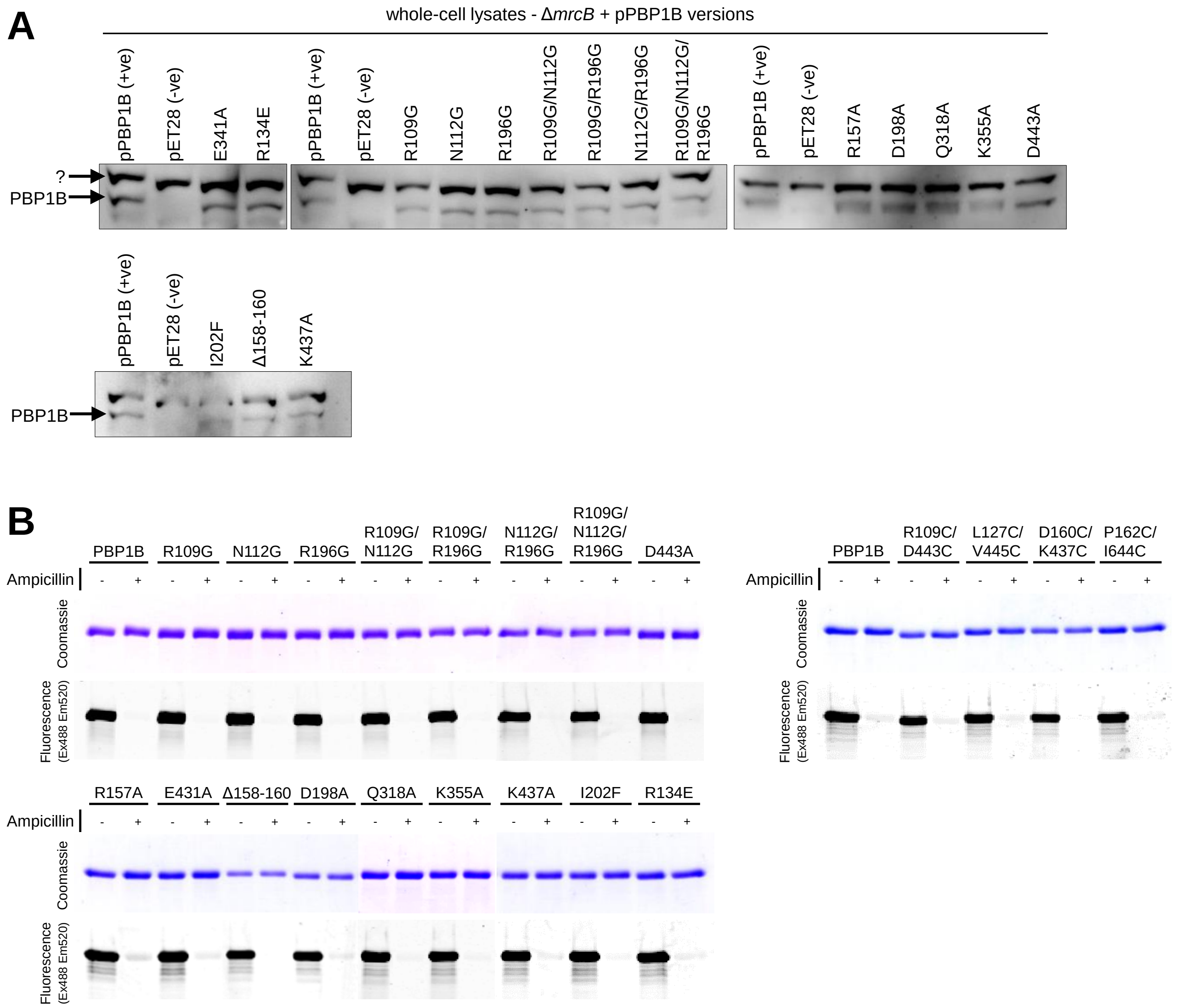

Supporting Figure 2: *In vivo* expression and Bocillin binding of PBP1B versions

A, Western blot detection of PBP1B versions expressed in BW25113Δ*mrcB*::FRT. Strains were grown to an OD_{578} of 0.5 before harvesting. Cells were lysed by addition of SDS-PAGE loading buffer and boiling at 100°C for 10 min. Total protein was resolved by SDS-PAGE, PBP1B was detected after western blot with specific α-PBP1B antibodies. An unknown additionally detected protein resolved just above PBP1B in the polyacrylamide gel.

B, Coomassie and fluorescence images of the same polyacrylamide gel in which purified PBP1B versions were resolved after binding to the fluorescent β-lactam Bocillin. As a control to show specificity of binding, ampicillin was incubated with each PBP1B version alongside an ampicillin-free duplicate at 37°C for 30 min. Its presence in samples indicated with a + above the lane. Bocillin was then added to all samples followed by further incubation at 37°C for 30 min. Binding of Bocillin shows the TPase domain, and presumably the entire protein is correctly folded and functional. Class A PBPs are notoriously unstable when attempts to truncate or significantly alter their domain architecture are made. Thus it is a fair assumption that any unfolding or gross structural instability of one portion would affect the entire molecule. This assay also serves to illustrate that each version does not undergo significant degradation at 37°C for 1 h.

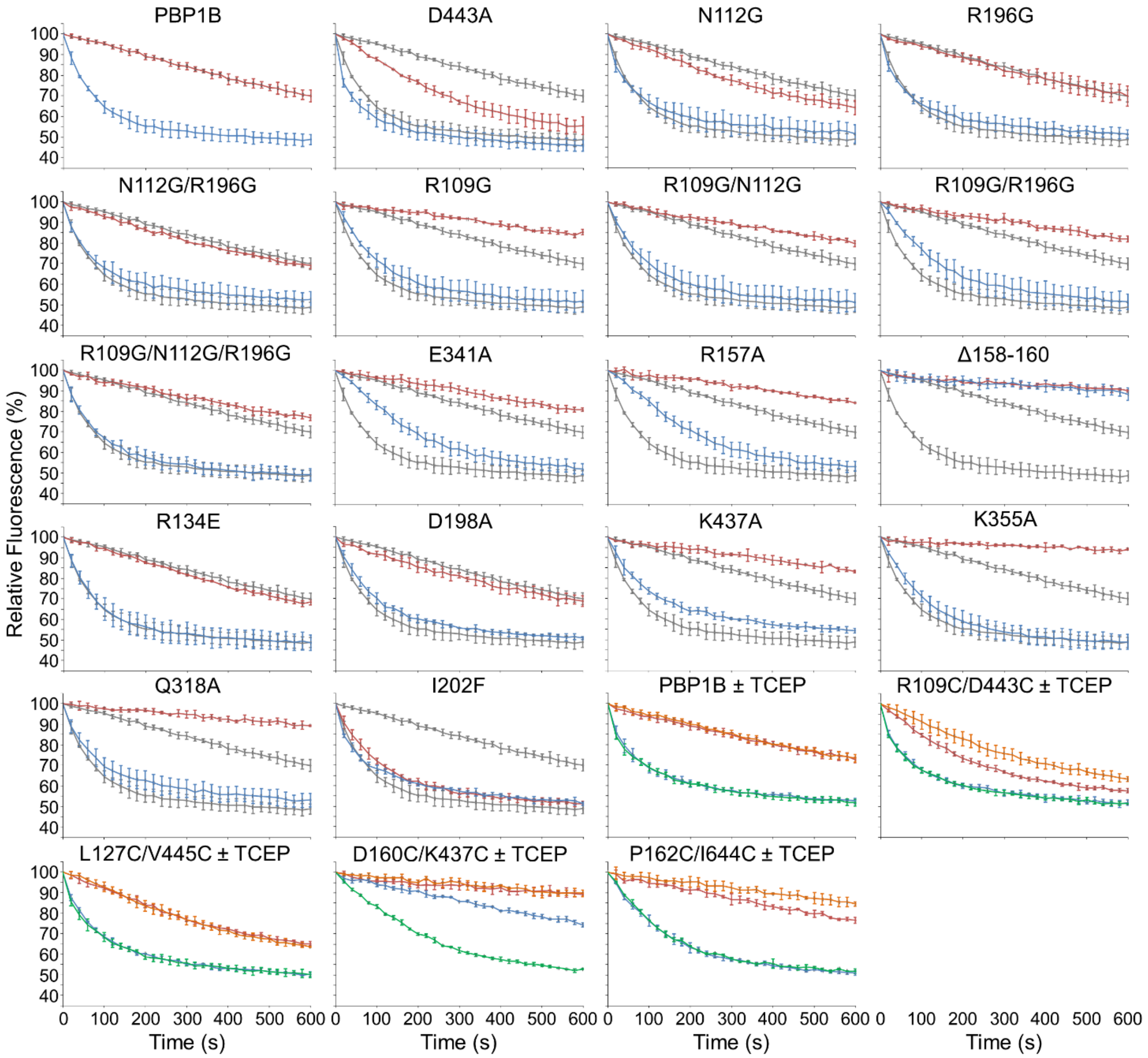

Supporting Figure 3: GTase activity data corresponding to Fig. 3A and 4B

Each individual graph shows the activity of a specific PBP1B version with and without LpoB (blue and red curves, respectively). For data corresponding to Fig. 3B each graph includes the WT activity curves, seen in the top left graph, in light grey for reference. Note that to ensure consistency and comparability between all PBP1B versions two independent WT PBP1B protein preparations were performed several months apart at the commencement of purification and towards the end. The WT PBP1B activity shown here is the mean of both preparations' activities. Curves with "± TCEP" in the label correspond to data shown in Fig. 4C and feature additional reactions performed at reducing conditions. For simplicity, these graphs no longer include the WT activity curves in grey. Red curve, disulphide or WT PBP1B version; blue curve, PBP1B version with LpoB; orange curve, PBP1B version in reducing conditions (+ TCEP); green curve, PBP1B version with LpoB in reducing conditions (+ TCEP). Each data point is the mean of 3 – 4 independent experiments ± SD.

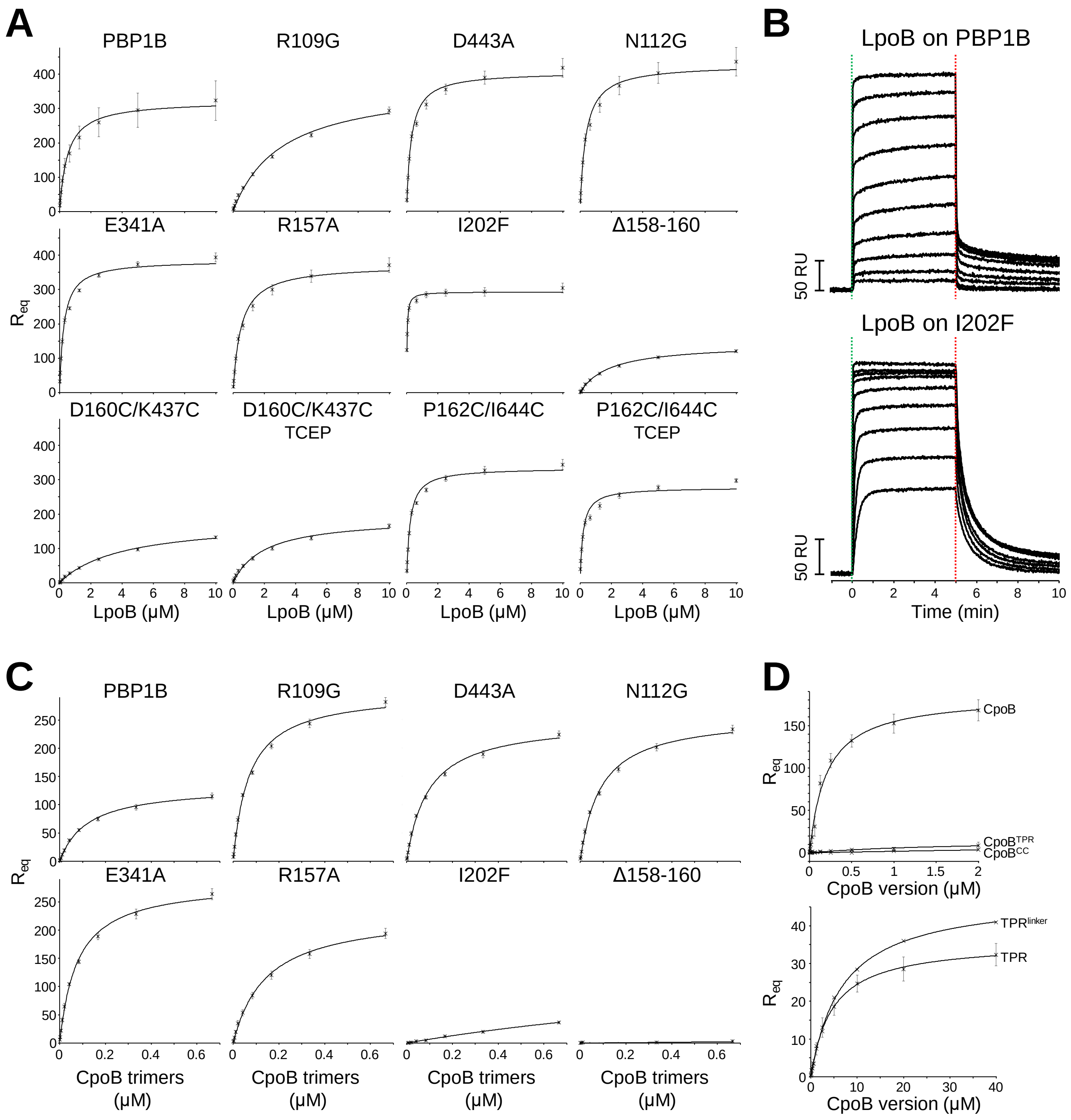

Supporting Figure 4: SPR interaction data

A, Average binding curves derived from injection of LpoB over PBP1B versions at concentrations ranging from 19.5 nM to 10 μM. Each point is the mean of 3 independent experiments ± SEM. Data points were fitted using SigmaPlot software with a one-site saturation ligand binding equation.

B, LpoB dissociates more slowly from $PBP1B^{I202F}$ compared to PBP1B, indicated by a greater area under the curves after the analyte injection was ceased. The red dashed line shows this point on representative SPR sensorgrams derived from LpoB (ranging from 19.5 nM to 10 μM) injection over PBP1B or $PBP1B^{I202F}$ surfaces. Injection initiated at time 0 (green dashed line).

C, Average binding curves derived from injection of CpoB over PBP1B versions at concentrations ranging from 3.9 nM to 2 μM, adjusted for CpoB trimerisation. Each point is the mean of 3 independent experiments ± SEM. Data points were fitted using SigmaPlot software with a one-site saturation ligand binding equation.

D, Average binding curves derived from injection of CpoB versions including WT CpoB, $CpoB^{TPR}$, $CpoB^{CC}$ (n = 3), and $CpoB^{linker}$ (n = 2). At standard analyte concentrations (3.9 nM to 2 μM) CpoB shows typical binding, the TPR domain minimal, and the CC domain none. Binding of the TPR domain is only reliably measurable at a higher analyte concentration range from 78 nM to 40 μM. The presence of the 30 a.a. linker sequence between the CC and TPR domains had no significant effect on $CpoB^{TPR}$ binding, with a K_D of 6.8 ± 0.7 μM compared to 4.9 ± 1.9 μM.

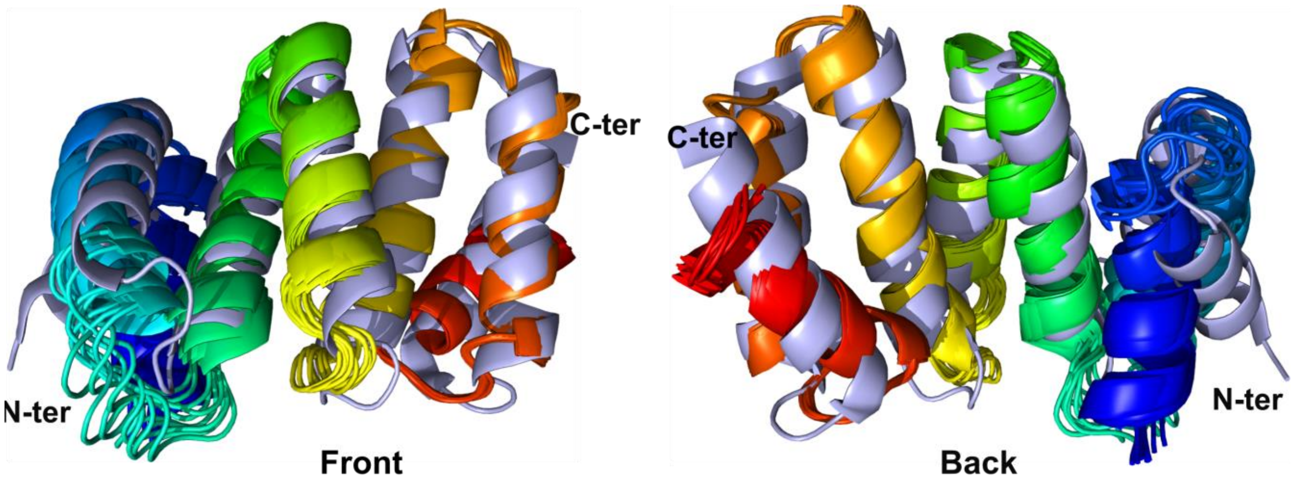

Supporting Figure 5: Structure of *E. coli* CpoB TPR domain

Cartoon representation of the NMR ensemble of the 20 lowest energy structures of *E. coli* CpoB coloured from blue (N-termini) to red (C-termini). The crystal structure of CpoB of *Xanthomonas campestris* (PDB 2XEV) was superimposed and coloured in light purple.

Supporting Table 1: NMR structures statistics

	Free UB2H	UB2H(-LpoB)	$CpoB^{TPR}$
**NMR Distances and dihedral constraints**			
Total unambiguous NOE	1610	1178	1926
Intra-residue [i = j]	502	310	414
Inter-residue	1108	868	1512
Sequential (|*i* - *j*| = 1)	418	346	508
Medium-range (|*i* - *j*| ≤ 1)	213	176	620
Long-range (|*i* - *j*| ≥ 1)	477	346	384
Total ambiguous NOE	214	259	860
Hydrogen bonds	0	0	0
Total dihedral-angle restraints			
Backbone φ	90	77	117
Backbone ψ	90	77	117
**Structure statistics**			
Average pairwise r.ms. deviation (Å) [a,*]			
Backbone atoms	0.8	1.5	0.7
All heavy atoms	1.2	1.8	1.1
Restraints violations [&,*]			
Distance > 0.5 Å	7.05	4.15	13.15
Dihedral (> 5º)	13.1	7.95	18.1
Ramachandran analysis [&,*]			
Residues in most favored regions (%)	79.3	80.6	89.0
Residues in additional allowed regions (%)	20.7	16.5	10.9
Residues in generously allowed regions (%)	0.0	2.8	0.1
Residues in disallowed regions (%)	0.0	0.1	0.0

[a] Pairwise r.m.s. deviation was calculated among the 20 refined structures of lowest energy.
[&] Average value, calculated on the 20 refined lowest energy structures, of the number of restraints violations.
* Values were calculated using the *Procheck* server (https://www.ebi.ac.uk/thornton-srv/software/PROCHECK/) Only residues in globular domains were considered in the reported statistics (residues 110-147, 150-191, and 194-199 for free UB2H; residues 116-166 and 168-189 for UB2H(-LpoB); residues 22 to 145 for $CpoB^{TPR}$).

Supporting Table 2: Bacterial strains

Name	Description	Ref.
BW25113	Wild-type strain and parent strain for Keio single-gene knockout library; genotype F- Δ(*araD-araB*)567 lacZ4787(del)::rrnB-3 LAM- rph-1 Δ(*rhaD-rhaB*)568 hsdR514.	Baba 2006
Δ*mrcB::kan*	*E. coli* KEIO collection BW25113 Δ*mrcB::kan*.	Baba 2006
Δ*mrcB*::FRT	Kan resistance cassette excised from BW25113Δ*mrcB::kan* using pCP20 Flp recombinase, leaving FRT-site scar in the genome.	This work
Δ*mrcA*::*cat*	*E. coli* ASKA collection BW38029 Δ*mrcA*::*cat*	unpublished; Mori H & colleagues
JE5615	Δ*mrcB mrcA*^{TS}.<br>Genotype; *ponA*^{TS}1104 *ponB*353 *dacA*1191 *dacB*12 *dapA lysA proA3 metB1 lac-3* Str^{r}.	Hirota
BL21 (DE3)	Expression strain for IPTG induced overproduction of proteins encoded by pET28a; genotype; F- *ompT*, *dcm*, *hsdS* (rB- mB-) *gal* λ(DE3)	Novagen
DH5α	Cloning and plasmid storage strain, genotype; *huA2, lacU169, phoA, glnV44, Φ80' lacZ, gyrA96, recA1, relA1, endA1, thi-1, hsdR17.*	Invitrogen

Supporting Table 3: Plasmids

Name	Description	Source/Ref
pET28a(+)	For IPTG inducible overproduction of encoded gene. KanR.	Novagen
pCpoB	Plasmid for overproduction of His$_6$-CpoB. Gene encoding *E. coli* CpoB res. 26 – 263 inserted between Nde I and Sac I sites of pET28a(+). Protein: His$_6$-Thrombin-CpoB$^{26\text{-}263}$.	Gray 2015
pAJFE01	Plasmid for overproduction of His$_6$-CpoB^{TPR}. Gene encoding *E. coli* CpoB res. 139 - 263 inserted between Nde I and Hind III sites of pET28a(+). Protein: His$_6$-Thrombin-CpoB$^{139\text{-}263}$.	This work
pAJFE02	Plasmid for overproduction of His$_6$-CpoB^{CC}. Gene encoding *E. coli* CpoB res. 27 - 109 inserted between Nde I and Hind III sites of pET28a(+). Protein: His$_6$-Thrombin-CpoB$^{27\text{-}109}$.	This work
pAJFE26	Plasmid for overproduction of His$_6$-CpoB$^{TPR+linker}$. Gene encoding *E. coli* CpoB res. 110 - 263 inserted between Nde I and Hind III sites of pET28a(+). Protein: His$_6$-Thrombin-CpoB$^{110\text{-}263}$.	This work
pAJFE03	Plasmid for overproduction of His$_6$-UB2H. Gene encoding PBP1B res. 108 - 200 inserted between Nde I and Hind III sites of pET28a(+). Protein: His$_6$-Thrombin-PBP1B$^{108\text{-}200}$.	This work
pLpoB^{sol}	Plasmid for overproduction of His$_6$-LpoB^{sol}. Gene encoding *E. coli* LpoB res. 21 - 213 inserted between Nde I and Hind III sites of pET28a(+). Protein: His$_6$-Thrombin-LpoB$^{21\text{-}213}$.	Typas 2010
pDML924	Plasmid for overproduction of PBP1Bγ. Gene encoding *E. coli* PBP1B res. 46 - 844 (PBP1Bγ) inserted between Nhe I and EcoR I sites of pET28a(+). Protein: His$_6$-Thrombin-PBP1B$^{46\text{-}844}$. Also referred to as pPBP1B in Fig. 2B.	Terrak 1999
pAJFE10	Plasmid for overproduction of His$_6$-PBP1By^{E341A}. Created by mutagenesis of pDML924. Also referred to as pE341A in Fig. 2B.	This work
pAJFE11	Plasmid for overproduction of His$_6$-PBP1By^{R109G}. Created by mutagenesis of pDML924. Also referred to as pR109G in Fig. 2B.	This work
pAJFE12	Plasmid for overproduction of His$_6$-PBP1By^{R196G}. Created by mutagenesis of pDML924. Also referred to as pR196G in Fig. 2B.	This work
pAJFE13	Plasmid for overproduction of His$_6$-PBP1By^{N112G}. Created by mutagenesis of pDML924. Also referred to as pN112G in Fig. 2B.	This work
pAJFE14	Plasmid for overproduction of His$_6$-PBP1By^{R134E}. Created by mutagenesis of pDML924. Also referred to as pR134E in Fig. 2B.	This work
pAJFE17	Plasmid for overproduction of His$_6$-PBP1By$^{N112G/R196G}$. Created by mutagenesis of pAJFE13. Also referred to as pN112G/R196G in Fig. 2B.	This work
pAJFE18	Plasmid for overproduction of His$_6$-PBP1By$^{R109G/R196G}$. Created by mutagenesis of pAJFE11. Also referred to as pR109G/R196G in Fig. 2B.	This work
pAJFE19	Plasmid for overproduction of His$_6$-PBP1By$^{R109G/N112G}$. Created by mutagenesis of pAJFE13. Also referred to as pR109G/N112G in Fig. 2B.	This work
pAJFE20	Plasmid for overproduction of His$_6$-PBP1By$^{R109G/N112G/R196G}$. Created by mutagenesis of pAJFE19. Also referred to as pR109G/N112G/R196G in Fig. 2B.	This work
pAJFE21	Plasmid for overproduction of His$_6$-PBP1By^{R157A}. Created by mutagenesis of pDML924. Also referred to as pR157A in Fig. 2B.	This work
pAJFE22	Plasmid for overproduction of His$_6$-PBP1By^{D198A}. Created by mutagenesis of pDML924. Also referred to as pD198A in Fig. 2B.	This work
pAJFE23	Plasmid for overproduction of His$_6$-PBP1By^{Q318A}. Created by mutagenesis of pDML924. Also referred to as pQ318A in Fig. 2B.	This work
pAJFE24	Plasmid for overproduction of His$_6$-PBP1By^{K355A}. Created by mutagenesis of pDML924. Also referred to as pK355A in Fig. 2B.	This work
pAJFE25	Plasmid for overproduction of His$_6$-PBP1By^{D443A}. Created by mutagenesis of pDML924. Also referred to as pD443A in Fig. 2B.	This work
pAJFE39	Plasmid for overproduction of His$_6$-PBP1By^{K437A}. Created by mutagenesis of pDML924. Also referred to as pK437A in Fig. 2B.	This work
pAJFE43	Plasmid for overproduction of His$_6$-PBP1By$^{\Delta 158\text{-}160}$. Created by mutagenesis of pDML924. Also referred to as pΔ158-160 in Fig. 2B.	This work
pAJFE45	Plasmid for overproduction of His$_6$-PBP1By^{I202F}. Created by mutagenesis of pDML924. Also referred to as pI202F in Fig. 2B.	This work
pAJFE32	Plasmid for overproduction of His$_6$-PBP1By$^{R109C/D443C}$. Created by mutagenesis of pDML924.	This work
pAJFE33	Plasmid for overproduction of His$_6$-PBP1By$^{L127C/V445C}$. Created by mutagenesis of pDML924.	This work
pAJFE36	Plasmid for overproduction of His$_6$-PBP1By$^{D160C/K437C}$. Created by mutagenesis of pDML924.	This work
pAJFE37	Plasmid for overproduction of His$_6$-PBP1By$^{P162C/I644C}$. Created by mutagenesis of pDML924.	This work

pCP20	Encoding Flp recombinase for the excision of FRT-flanked KanR cassettes. CmR, AmpR, temperature sensitive replicon.	Cherepanov 1995

Supporting Table 4: Primers

Name	DNA sequence	Description
CpoB_TPR Fwd.	ATATACATATGAGCGGTAATGCAAACACG	ATATA-[NdeI]-[*cpoB* b.415 forward]. Used to make pAJFE01.
CpoB_TPR Rev.	TATATAAGCTTTTACATCGCGTTCAGACG	TATAT-[HindIII]-[*cpoB* stop reverse]. Used to make pAJFE01 and pAJFE26
CpoB_CC Fwd.	ATATACATATGCAGGCACCAATCAGTAGTGTC	ATATA-[NdeI]-[*cpoB* b.79 forward]. Used to make pAJFE02.
CpoB_CC Rev.	TATATAAGCTTTTATTGCGCCGCTGC	TATAT-[HindIII]-[*cpoB* b.327 reverse]. Used to make pAJFE01.
CpoB_TPR+link Fwd.	ATATACATATGTCAACCAGCGGCGATCAAAG	ATATA-[NdeI]-[*cpoB* b.328 forward]. Used to make pAJFE26.
UB2H Fwd.	ATATACATATGGGCCGAATGGTCAATCTT	ATATA-[NdeI]-[*mrcB* b.460 forward]. Used to make pAJFE03.
UB2H Rev.	TATATAAGCTTTTAACGCGGATCAAGACGGAA	TATAT-[HindIII]-[*mrcB* b.738 reverse]. Used to make pAJFE03.
1B-E341A Fwd.	CGCCCGGTAGAAGCGCTAAGCCTCGAC	Mutagenesis primer to introduce Ala in place of Glu341 in PBP1B.
1B-E341A Rev.	GTCGAGGCTTAGCGCTTCTACCGGGCG	Mutagenesis primer to introduce Ala in place of Glu341 in PBP1B.
1B-R109G Fwd.	CTGCGGCAGTTTATGGCGGAATGGTCAATCTTGAG	Mutagenesis primer to introduce Gly in place of Arg109 in PBP1B.
1B-R109G Rev.	CTCAAGATTGACCATTCCGCCATAAACTGCCGCAG	Mutagenesis primer to introduce Gly in place of Arg109 in PBP1B.
1B-R109G II Fwd.	GCGGCAGTTTATGGCGGAATGGTCGGTCTTG	Mutagenesis primer to introduce Gly in place of Arg109 in PBP1B^{N112G}.
1B-R109G II Rev.	CAAGACCGACCATTCCGCCATAAACTGCCGC	Mutagenesis primer to introduce Gly in place of Arg109 in PBP1B^{N112G}.
1B-R196G Fwd.	CAGTTCGGTTTCTTCGGTCTTGATCCGCGTC	Mutagenesis primer to introduce Gly in place of Arg196 in PBP1B.
1B-R196G Rev.	GACGCGGATCAAGACCGAAGAAACCGAACTG	Mutagenesis primer to introduce Gly in place of Arg196 in PBP1B.
1B-N112G Fwd.	AGTTTATGGCCGAATGGTCGGTCTTGAGCCAGACATGACC	Mutagenesis primer to introduce Gly in place of Asn112 in PBP1B.
1B-N112G Rev.	GGTCATGTCTGGCTCAAGACCGACCATTCGGCCATAAACT	Mutagenesis primer to introduce Gly in place of Asn112 in PBP1B.
1B-R134E pt.1 Fwd.	GCTGGAGGCGACCCAGTATGATCAGGTGTCG	Mutagenesis primer to introduce 2 of 3 required base changes for Arg134 to Glu in PBP1B.
1B-R134E pt.1 Rev.	CGACACCTGATCATACTGGGTCGCCTCCAGC	Mutagenesis primer to introduce 2 of 3 required base changes for Arg134 to Glu in PBP1B.
1B-R134E pt.2 Fwd.	GAGGCGACCCAGTATGAGCAGGTGTCGAAAATGAC	Mutagenesis primer to complete Arg134 to Glu in PBP1B.

1B-R134E pt.2 Rev.	GTCATTTTCGACACCTGCTCATACTGGGTCGCCTC	Mutagenesis primer to complete Arg134 to Glu in PBP1B.
1B-R157A Fwd.	GCATTGAGATGATTCGCGCTCCGTTTGATTTCCCGG	Mutagenesis primer to introduce Ala in place of Arg157 in PBP1B.
1B-R157A Rev.	CCGGGAAATCAAACGGAGCGCGAATCATCTCAATGC	Mutagenesis primer to introduce Ala in place of Arg157 in PBP1B.
1B-D198A Fwd.	GGTTTCTTCCGTCTTGCTCCGCGTCTGATCACC	Mutagenesis primer to introduce Ala in place of Asp198 in PBP1B.
1B-D198A Rev.	GGTGATCAGACGCGGAGCAAGACGGAAGAAACC	Mutagenesis primer to introduce Ala in place of Asp198 in PBP1B.
1B-Q318A Fwd.	GAGGTGTATCTCGGTGCGAGCGGCGACAACGA	Mutagenesis primer to introduce Ala in place of Gln318 in PBP1B.
1B-Q318A Rev.	TCGTTGTCGCCGCTCGCACCGAGATACACCTC	Mutagenesis primer to introduce Ala in place of Gln318 in PBP1B.
1B-K355A Fwd.	CTGTTAGTCGGTATGGTGGCAGGGGCGTCCATCTACAA	Mutagenesis primer to introduce Ala in place of Lys355 in PBP1B.
1B-K355A Rev.	TTGTAGATGGACGCCCCTGCCACCATACCGACTAACAG	Mutagenesis primer to introduce Ala in place of Lys355 in PBP1B.
1B-D443A Fwd.	CTTCACTACCTTTGCCTCGGTGGCCCAGG	Mutagenesis primer to introduce Ala in place of Asp443 in PBP1B.
1B-D443A Rev.	CCTGGGCCACCGAGGCAAAGGTAGTGAAG	Mutagenesis primer to introduce Ala in place of Asp443 in PBP1B.
1B-R109C pt.1 Fwd.	CTGCGGCAGTTTATGGCTGAATGGTCAATCTTGAG	Mutagenesis primer to introduce 1st base change for Arg109 to Cys in PBP1B.
1B-R109C pt.1 Rev.	CTCAAGATTGACCATTCAGCCATAAACTGCCGCAG	Mutagenesis primer to introduce 1st base change for Arg109 to Cys in PBP1B.
1B-R109C pt.2 Fwd.	GCGGCAGTTTATGGCTGCATGGTCAATCTTGAGCC	Mutagenesis primer to complete Arg109 to Cys in PBP1B.
1B-R109C pt.2 Rev.	GGCTCAAGATTGACCATGCAGCCATAAACTGCCGC	Mutagenesis primer to complete Arg109 to Cys in PBP1B.
1B-D443C Fwd.	GGCGTGAAGATCTTCACTACCTTTTGCTCGGTGGCCCAG	Mutagenesis primer to introduce Cys in place of Asp443 in PBP1B^{R109C}.
1B-D443C Rev.	CTGGGCCACCGAGCAAAAGGTAGTGAAGATCTTCACGCC	Mutagenesis primer to introduce Cys in place of Asp443 in PBP1B^{R109C}.
1B-L127C pt.1 Fwd.	GAACGAGATGGTGAAGTGGCTGGAGGCGACCCAG	Mutagenesis primer to introduce 2 of 3 required base changes for Leu127 to Cys in PBP1B.
1B-L127C pt.1 Rev.	CTGGGTCGCCTCCAGCCACTTCACCATCTCGTTC	Mutagenesis primer to introduce 2 of 3 required base changes for Leu127 to Cys in PBP1B.
1B-L127C pt.2 Fwd.	AGATGGTGAAGTGCCTGGAGGCGACCC	Mutagenesis primer to complete Leu127 to Cys in PBP1B.
1B-L127C pt.2 Rev.	GGGTCGCCTCCAGGCACTTCACCATCT	Mutagenesis primer to complete Leu127 to Cys in PBP1B.
1B-V445C pt.1 Fwd.	CTACCTTTGACTCGTGGGCCCAGGACGCGG	Mutagenesis primer to introduce 2 of 3 required base changes for Val445 to Cys in PBP1B^{L127C}.
1B-V445C pt.1 Rev.	CCGCGTCCTGGGCCCACGAGTCAAAGGTAG	Mutagenesis primer to introduce 2 of 3 required base

		changes for Val445 to Cys in PBP1B^{L127C}.
1B-V445C pt.2 Fwd.	CTTTGACTCGTGCGCCCAGGACGCG	Mutagenesis primer to complete Val445 to Cys in PBP1B^{L127C}.
1B-V445C pt.2 Rev.	CGCGTCCTGGGCGCACGAGTCAAAG	Mutagenesis primer to complete Val445 to Cys in PBP1B^{L127C}.
1B-D160C Fwd.	CATTGAGATGATTCGCCGTCCGTTTTGTTTCCCGGACAGTA	Mutagenesis primer to introduce Cys in place of Asp160 in PBP1B.
1B-D160C Rev.	TACTGTCCGGGAAACAAAACGGACGGCGAATCATCTCAATG	Mutagenesis primer to introduce Cys in place of Asp160 in PBP1B.
1B-K437C Fwd.	CGAGTCAAAGGTAGTGAAGATGCACACGCCGGAGAGATCTTTTAC	Mutagenesis primer to introduce Cys in place of Lys437 in PBP1B^{D160C}.
1B-K437C Rev.	GTAAAAGATCTCTCCGGCGTGTGCATCTTCACTACCTTTGACTCG	Mutagenesis primer to introduce Cys in place of Lys437 in PBP1B^{D160C}.
1B-P162C Fwd.	CCTGTCCTTCTTTACTGTCGCAGAAATCAAACGGACGGCGA	Mutagenesis primer to introduce Cys in place of Pro162 in PBP1B.
1B-P162C Rev.	TCGCCGTCCGTTTGATTTCTGCGACAGTAAAGAAGGACAGG	Mutagenesis primer to introduce Cys in place of Pro162 in PBP1B.
1B-I644C Fwd.	CCATCTTCCGCGCATACCGAACGCAGCGCAGAAAG	Mutagenesis primer to introduce Cys in place of Ile644 in PBP1B^{P162C}.
1B-I644C Rev.	CTTTCTGCGCTGCGTTCGGTATGCGCGGAAGATGG	Mutagenesis primer to introduce Cys in place of Ile644 in PBP1B^{P162C}.
1B-K437A Fwd.	AAAAGATCTCTCCGGCGTGGCGATCTTCACTACCTTTGAC	Mutagenesis primer to introduce Ala in place of Lys437 in PBP1B.
1B-K437A Rev.	GTCAAAGGTAGTGAAGATCGCCACGCCGGAGAGATCTTTT	Mutagenesis primer to introduce Ala in place of Lys437 in PBP1B.
1BΔ158-160 Fwd.	CATTGAGATGATTCGCCGTTTCCCGGACAGTAAAGAAG	Mutagenesis primer to excise coding bases for res.158 to 160 in the PBP1B sequence.
1BΔ158-160 Rev.	CTTCTTTACTGTCCGGGAAACGGCGAATCATCTCAATG	Mutagenesis primer to excise coding bases for res.158 to 160 in the PBP1B sequence.
1B-I202F Fwd.	GTCTTGATCCGCGTCTGTTCACCATGATCTCTTCG	Mutagenesis primer to introduce Phe in place of Ile202 in PBP1B.
1B-I202F Rev.	CGAAGAGATCATGGTGAACAGACGCGGATCAAGAC	Mutagenesis primer to introduce Phe in place of Ile202 in PBP1B.
1B-seq F	AGGGCATCAGCGCGCACGTT	Forward primer to amplify from 150 bp upstream of *mrcB* gene in *E. coli*
1B-seq R	TCGAAACGCTGGCGGGTTCG	Reverse primer to amplify from 150 downstream of *mrcB* gene in *E. coli*
1A-seq F	GCAAACCAATTTGCCAGATC	Forward primer to amplify from 150 bp upstream of *mrcA* gene in *E. coli*
1A-seq R	CTGATGCACAATGTTTATC	Reverse primer to amplify from 150 downstream of *mrcA* gene in *E. coli*
